# An antibody targeting type III secretion system induces broad protection against *Salmonella* and *Shigella* infections

**DOI:** 10.1371/journal.pntd.0009231

**Published:** 2021-03-12

**Authors:** Raphaël Sierocki, Bakhos Jneid, Maria Lucia Orsini Delgado, Marc Plaisance, Bernard Maillère, Hervé Nozach, Stéphanie Simon

**Affiliations:** 1 Université Paris Saclay, CEA, INRAE, Département Médicaments et Technologies pour la Santé (DMTS), SIMoS, Gif-sur-Yvette, France; 2 Université Paris Saclay, CEA, INRAE, Département Médicaments et Technologies pour la Santé (DMTS), SPI, Gif-sur-Yvette, France; Instituto Butantan, BRAZIL

## Abstract

*Salmonella* and *Shigella* bacteria are food- and waterborne pathogens that are responsible for enteric infections in humans and are still the major cause of morbidity and mortality in the emerging countries. The existence of multiple *Salmonella* and *Shigella* serotypes as well as the emergence of strains resistant to antibiotics requires the development of broadly protective therapies. Recently, the needle tip proteins of the type III secretion system of these bacteria were successfully utilized (SipD for *Salmonella* and IpaD for *Shigella*) as vaccine immunogens to provide good prophylactic cross-protection in murine models of infections. From these experiments, we have isolated a cross-protective monoclonal antibody directed against a conserved region of both proteins. Its conformational epitope determined by Deep Mutational Scanning is conserved among needle tip proteins of all pathogenic *Shigella* species and *Salmonella* serovars, and are well recognized by this antibody. Our study provides the first *in vivo* experimental evidence of the importance of this common region in the mechanism of virulence of *Salmonella* and *Shigella* and opens the way to the development of cross-protective therapeutic agents.

## Author summary

*Salmonella* and *Shigella* are responsible for gastrointestinal diseases and continue to remain a serious health hazard in South and South-East Asia and African countries, even more with the new emergence of multi drug resistances. Developed vaccines are either not commercialized (for *Shigella*) or cover only a limited number of serotypes (for *Salmonella*). There is thus a crucial need to develop cross-protective therapies. By targeting proteins SipD and IpaD belonging respectively to the injectisome of *Salmonella* and *Shigella* and necessary to their virulence, we have shown that a monoclonal antibody (mAb) directed against a conserved common region of their apical part provides good cross-protection prophylactic efficacy. We have determined the region targeted by this mAb which could explain why it is conserved among *Salmonella* and *Shigella* bacteria.

## Introduction

*Salmonella* and *Shigella* are Gram-negative enteropathogenic bacteria belonging to the Enterobacteriaceae family [1,2] and responsible for gastrointestinal diseases. They continue to remain a serious health hazard in South and South-East Asia and African countries [3–7], causing notably severe diarrhea in children under the age of five in sub-Saharan Africa and south Asia [8–10]. Other at-risk populations include military personnel deployed abroad [11–13], travelers, and victims of bioterrorist attacks [14,15]. While *Salmonella* and *Shigella* consist of few species (two for *Salmonella*: *S*. *enterica* and *S*. *bongori* and four for *Shigella*: *S*. *flexneri*, *S*. *sonnei*, *S*. *dysenteriae* and *S*. *boydii*), there is a multiplicity of subspecies [16–18], which makes it difficult to develop broad range vaccines. Moreover, active immune system stimulation induced by vaccination takes days to weeks to be effective and can only be used to prevent infections. As opposed to active immunotherapy, passive immunotherapy provides immediate protection and is more suitable for therapeutic purposes. Although rarely utilized for infectious diseases, and particularly for bacterial diseases for which there are other therapeutic solutions, the emergence and rapid spread of bacterial strains resistant to multiple antibiotics [19] might change this situation. As Type 3 Secretion System (T3SS) is essential for virulence and is conserved among all pathogenic *Salmonella* and *Shigella* strains [20], T3SS proteins appear to be ideal candidates for broad *Salmonella-Shigella* vaccine [21] and passive immunotherapy development.

Components of T3SSs are widely distributed in gram-negative pathogens and are well conserved with regard to their overall structure, architecture, and function, and especially in *Salmonella*, *Shigella* and *Burkholderia*. These injectisomes are composed of a basal body that traverses the inner and outer bacterial membrane and a needle-like complex that emerges at its apical end. The needle-tip is formed by a multimeric hydrophilic protein complex (including IpaD for *Shigella* [22–28] and SipD for *Salmonella* [29–31]) connecting the bacterium to the host membrane through the needle [32–34] through which effectors are secreted [35]. The tip proteins IpaD and SipD assemble at the distal end of the needle and form a pentameric ring that prevents premature secretion of effectors until host cell sensing and/or exposure to small molecules such as bile salts [23,36–41], which trigger a structural rearrangement and recruitment of the hydrophobic translocator protein IpaB [37] for *Shigella* and SipB [42] for *Salmonella* to the tip complex to prepare host membrane interaction. Upon contact with host membrane, the second hydrophobic translocator IpaC for *Shigella* or its counterpart SipC for *Salmonella* is recruited to the tip of the T3SS, thereby promoting translocon pore formation and induction of effector protein secretion into the host cell followed by pathogen invasion [43].

Multiple models have been proposed for the structure of the tip complex and all agree on the formation of a pentamer at the distal end of the needle containing at least four molecules of IpaD [22,24,26,41,44]. The fifth member of the pentamer is either another IpaD molecule or the first translocator protein IpaB and no data are available regarding SipD. The most recent electron microscopy study of nascent apparatuses conducted by Blocker and co-workers hypothesizes that active tip complexes carry one IpaB for four IpaD, which fits the electron map densities of tip complexes [44]. They showed that homopentameric and heteropentameric tip complexes coexist in wild-type strains, hypothesizing that the heteropentamer is the active state of the complex and that one IpaB replaces one IpaD in the pentamer after the activation signal. As the complete structure of IpaB is not yet known, although it has recently been partially solved by Barta *et al* [45], Blocker and co-workers proposed a structure of 4 IpaD lacking the fifth subunit supposed to be IpaB (PDB #4D3E) [44].

During infection, the bacteria receive an external signal from the host environment and begin to assemble coordinately the constituents of the secretion system [46,47] which ultimately leads to the injection of effectors and/or invasion of the targeted host cell by the bacterium [48–53]. Based on the literature and our results, the needle-tip proteins have proved to be immunogenic in mice and in humans, able to elicit good humoral responses protective against salmonellosis and shigellosis [54–58]. Moreover, the sequence identity between IpaD and SipD [59], led us to the hypothesis that those needle-tip proteins might be suitable targets for the development of cross-protective immunity against *Shigella*/*Salmonella*.

With this aim, a murine monoclonal antibody (mAb) recognizing both IpaD and SipD was generated and evaluated *in vivo*. Cross-protective efficacy was determined against lethal oral intestinal infection with 100 LD50 of *Salmonella enteritidis* serovar Typhimurium or lethal intranasal infection with 100 LD50 of *Shigella flexneri 2a*. We provide the first demonstration that a monoclonal anti-IpaD/SipD antibody is protective in prophylaxis against *Salmonella* and *Shigella* infections in a murine model. Moreover, this antibody increases contact-mediated hemolysis *in vitro* and partially blocks the invasion of cultured cells by *Shigella*, confirming that the two mechanisms, already identified as carried by two distinct domains of IpaD, can be mechanistically dissociated [60]. Determination of its epitope of recognition sheds new light on the role of the needle tips proposed in the literature on the basis of structural models.

## Methods

### Ethics statement

Six- to 8-week-old female BALB/c mice from Janvier Labs, France were maintained in accordance with French and European regulations on care and protection of laboratory animals (European Community [EC] Directive 2010/63/UE, French Law 2001–486, 6 June 2001) and with the agreement of the ethics committee (CETEA) no. 15–055 delivered to S. Simon and agreement D-91-272-106 from the Veterinary Inspection Department of Essonne (France). Up to ten mice were kept in each cage and housed in a temperature-regulated-room and had free access to food and water. All animal experiments minimized suffering in line with the guideline of the CETEA committee.

### Bacterial strains

The *Salmonella enterica* serovar Typhimurium (CIP 104474, Pasteur Institute collection) and *Shigella flexneri 2a* (generous gift from Dr A. Phalipon, Pasteur Institute) were used in this study. Bacteria were first grown at 37°C on agar plates (trypticase soy (TCS) containing 0.01% Congo red (Serva) *for S*. *flexneri 2a* and LB plates *for S*. Typhimurium). For infection, a colony (Congo red-positive for *S*. *flexneri* 2a) was picked for a 5ml overnight (O/N) culture at 37°C in LB medium, followed by a culture in the same medium with 1:100 of the first culture for 2 h under the same conditions.

### Reagents

Biotin N-hydroxysuccinimide ester and streptavidin were from Sigma-Aldrich. Goat anti-mouse IgG and IgM polyclonal antibodies were from Jackson ImmunoResearch (Interchim, France). ELISAs were performed with MaxiSorp 96-well microtiter plates (Nunc, Thermoscientific), and all reagents were diluted in enzyme immunoassay (EIA) buffer (0.1 M phosphate buffer [pH 7.4] containing 0.15 M NaCl, 0.1% bovine serum albumin [BSA], and 0.01% sodium azide).

### Production of monoclonal antibodies

The *sipD* and *ipaD* genes from respectively *S*. Typhimurium and *S*. *flexneri* were synthesized (Genecust) based on the published sequences of *Salmonella* strain CIP 104474 and of *Shigella* strain CIP 82.48T and cloned into *Nde*I/*Xho*I restriction sites of the IPTG inducible pET22b vector (Novagen), allowing insertion of a poly-histidine tag sequence at the 3′ end of the genes. Whole proteins SipD and IpaD were expressed and purified by affinity chromatography (Ni-NTA) as described previously [61]. Six- to 8-week-old female BALB/c mice were immunized 3 times with recombinant His-tagged IpaD either by the intranasal route (10 μg of recombinant protein in 30 μL, with cholera toxin as adjuvant) or by the subcutaneous route (20 μg of recombinant protein in 100 μLwith alum hydroxide as adjuvant). Mice were bled before the first immunization (P0, used as the negative control) and 2 weeks after each injection (P1, P2 and P3). The immune polyclonal response was evaluated by enzyme immunoassay (EIA) using goat anti-mouse Ig(G+M) as coated antibodies (see "Enzyme immunoassays" below). The two mice presenting the highest immune response (one immunized intranasally and one subcutaneously, S1 Fig) were selected for preparation of mAbs and given three intravenous booster injections of IpaD recombinant protein 2 months after the last immunization. Two days after the last boost, spleen cells from mice were fused with myeloma NS1 cells as previously described [62,63]. The hybridoma culture supernatants were screened for the presence of anti-IpaD/SipD antibodies (that is to say antibodies recognizing both proteins) by EIA (see below). Selected hybridomas were subsequently cloned by limiting dilution. MAbs were further purified by affinity chromatography using protein A and dialyzed in 0.05 M phosphate buffer (pH 7.4). Purity was assessed by SDS PAGE and Coomassie blue staining.

### Production of VHHs

The amino-acid sequences of VHH-JMK-H2 and VHH-20ipaD were extracted from a previously published work by Barta *et al*. [28]. These two VHHs were produced in *E*.*coli* BL21 using a 6His-DsbC-TEV fusion as previously described for DsbC-DRPs (Disulfide-rich proteins) production [64]. The protocol used here differs after the TEV protease cleavage step: the cleaved VHHs were loaded onto a 5 mL HisTrap FF column (at a flow rate of 1 mL/min) and the flow-through was harvested. The cleaved VHHs were then loaded onto a preparative gel filtration column (HiPrep 16/60 Sphacryl S-100 High Resolution, GE-Healthcare). The fractions containing monomeric VHHs were pooled and concentrated using a centrifugal concentrator with a cut-off of 3000 Da (Vivaspin 20 3,000 MWCO PES membrane, Sartorius). Correct protein size and purity were confirmed by mass spectrometry and SDS-PAGE analysis.

### Evaluation of the cell invasion neutralizing activity of anti-IpaD/SipD Abs *in vitro*

Cell invasion test was adapted from a protocol previously described by Roehrich *et al* [41]. HeLa cells were distributed at a ratio of 3x10^5^ cells/well in a 24-well plate and cultured overnight in complete DMEM (high-glucose Dulbecco’s modified Eagle medium (DMEM) (Gibco), supplemented with 10% fetal calf serum, 1mM pyruvate, 2mM Glutamine, 1% MEM non-essential amino acids, 1% penicillin-streptomycin (Sigma)), in a humidified 5% CO_2_ atmosphere. *Shigella flexneri 2a* bacteria from overnight pre-culture were grown in CASO broth (Sigma) until OD_600_ ~ 1 (exponential growth), centrifuged (4500 x g, 10 minutes) and resuspended in PBS at 4.5x10^9^ bacteria/mL. HeLa cells were washed twice with 1mL PBS. One milliliter of complete DMEM-20mM HEPES, without penicillin-streptomycin, containing 10 μL of bacterial suspension (i.e. multiplicity of infection [MOI] of 100) and 500 nM final concentration of mAbs (IpaD-318 or Isotype control IgG1) or VHH (VHH-JMK-H2 or VHH-20ipaD), was added to each well. Control IgG1 was used as reference of 100% invasion. The plate was centrifuged (900 x g, 10 minutes) and incubated for 30 minutes at 37°C. The supernatants were then removed, cells washed four times with 1mL PBS, and 1mL of complete DMEM supplemented with 100 μg/mL gentamicin (Gibco) was added to cells for a further incubation of 2 h in a humidified 5% CO_2_ atmosphere. After removal of the culture medium, cells were lysed by addition of 1mL of 0.1% Triton X-100—PBS solution to each well, and incubation for 10 minutes at room temperature. Serial dilutions were made, 100 μL of each dilution plated on TCS agar plates, and colonies counted the next day.

### Contact-mediated hemolysis

The contact-mediated hemolysis assay has been slightly adapted from a previously described protocol [41]. *Shigella flexneri 2a* bacteria grown overnight in LB medium, were diluted in tubes containing 5mL of LB medium to a final OD_600_ of 0.05, and mAbs (IpaD-318 or Isotype control IgG1) or VHH (VHH-JMK-H2 or VHH-20ipaD) were added at a 1μM final concentration. Bacteria were then grown at 37°C during 4 to 5h until OD_600_ of approximately 1.5 to 2, collected by centrifugation at 5000 x g for 7 min at 4°C, and resuspended in PBS at a concentration of 10^10^ bacteria/mL. In parallel sheep red blood cells (RBCs, supplier Innovative Research) were washed three times in PBS by centrifugation at 2000 x g for 5 min at 4°C and resuspended at a final concentration of 5 x 10^8^ cells/mL. Then 100 μL of bacteria from each condition were distributed in round-bottom 96-well plates in addition to 100 μL of the RBC suspension and 100 μL of the mAbs or VHH at a final concentration of 3 μM were added. In control wells, bacteria and antibodies were replaced by 200 μL of ultrapure water (100% hemolysis) or PBS (baseline hemolysis control). Plates were centrifuged at 1500 x g for 10 min at 10°C and incubated at 37°C for 1h. After incubation, RBCs were resuspended, and the plates centrifuged at 2000 x g for 10 min at 4°C. After centrifugation, 100 μL of supernatant was transferred to a flat-bottom 96-well plate, and optical density at 540 nm was measured.

### Evaluation of the neutralizing activity of anti-IpaD/SipD mAbs *in vivo*

One day after administration of anti-IpaD/SipD mAbs (300 μg/mouse) by the intraperitoneal route, separately or in combination (for IpaD-301 + IpaD-318, 150 μg of each antibody/mouse), mice were challenged intragastrically (IG) (for *S*. Typhimurium) or intranasally (IN) (for *S*. *flexneri*) with bacteria at 100 LD50 (2x10^4^ CFU/mouse for *S*. Typhimurium and 10^9^ CFU/mouse for *S*. *flexneri 2a*, in agreement with previous publication using this strain [65]). The mAbs were also injected into mice after challenge and at onset of signs (5 days and 24 h after challenge for *S*. Typhimurium and *S*. *flexneri 2a* respectively). Mice administered PBS or control IgG were used as controls. Challenged mice were monitored daily for body weight loss and any signs of sickness. Mice that were considered to have reached the experimental endpoint were euthanized. The number of mice surviving after 30 days of daily observation was used to determine the relative degree of protection.

### Enzyme immunoassays

i) Labeling with biotin.

One hundred μg of recombinant proteins (SipD or IpaD) in 400 μL borate buffer (0.1 M; pH 8.5) was incubated at a 1:20 molar ratio with biotin-N-hydroxysuccinimide ester dissolved in 6 μL of anhydrous dimethylformamide (DMF). The reaction was stopped after 30 min at RT by adding 100 μL of 1 M Tris-HCl (pH 8) for 30 min. Finally, 500 μL of EIA buffer was added and the preparation was stored frozen at -20°C until use.

ii) Evaluation of polyclonal response, and screening of mAbs in hybridoma supernatants.

Anti-IpaD/SipD antibodies were measured in sera of immunized mice or hybridoma culture supernatants using sandwich ELISA. Briefly, microtiter plates were coated with 100 μL of goat anti-mouse Ig(G+M) at 10 μg/mL (diluted in 50 mM potassium phosphate buffer) overnight at RT. Plates were then saturated overnight at 4°C with 300 μL/well of EIA buffer. After a washing cycle performed with the washing buffer (0.01 M potassium phosphate [pH 7.4] containing 0.05% Tween 20), the plates were incubated with 100 μL/well of each hybridoma culture supernatant or serial dilutions of mouse sera (from 10^−2^ to 10^−5^) were added in duplicate and incubated overnight at 4°C (100 μL/well). The plates were then washed 3 times before adding 100 μL/well of biotinylated recombinant SipD or IpaD proteins at 100 ng/mL. After 2 hours of incubation at RT followed by three washing cycles, 100 μL/well of acetylcholinesterase (AChE; EC 3.1.1.7)-labeled streptavidin (1 Ellman unit/mL) was added and incubated for 1 hour at RT. Finally, the plates were washed 3 times and the absorbance was measured at 414 nm after 45 min of reaction with 200 μL/well of Ellman’s reagent [66].

### Western blot experiment

SipD or IpaD recombinant proteins were suspended in Laemmli buffer (containing 0.25 M Tris-HCl pH 6.8, 4% SDS, 40% glycerol, 0.1% bromophenol blue and 10% β-mercaptoethanol) and denatured for 5 min at 95°C. After migration of 10 ng and 100 ng/well of recombinant SipD or IpaD in SDS-PAGE for 1 h 30 min at 150 V in a 15% resolving gel, proteins were blotted onto a PVDF membrane (Amersham Biosciences) overnight at RT at 30 V. For the saturation step, the membranes were saturated with PBS containing 0.1% Tween 20 (PBST) and 5% skimmed dry milk for 30 min at RT. After two washes in PBST, specific mAbs (4 μg/mL in PBST containing 1% skimmed dry milk) were incubated for 30 min at RT with the membranes. After three washes in PBST, the membranes were reacted for 30 min at RT with HRP-labeled polyclonal goat anti-mouse immunoglobulins (Pierce) diluted to 1:2000 in PBST containing 3% skimmed dry milk. After three washes in PBST and a brief wash in PBS, bands were detected via chemiluminescence (ECL, Amersham Biosciences) using a VersaDoc imaging system (Bio-Rad).

### Statistical analysis

PRISM software v.5.04 (GraphPad Software inc., San Diego, CA) was used for graphics generation and statistical analyses. Survival rates were analyzed using the log-rank (Mantel-Cox) test. Data are presented as the mean ± standard errors SEM for 10 mice per group. A P value < 0.05 was considered significant in all determinations.

### Library design & generation

Two libraries of *ipaD* variants with single amino acid mutations were generated using the “plasmid one pot saturation mutagenesis” method of Wrenbeck *et al* [67]. A library was generated for each of the two *ipaD* regions targeted (library 1: amino acids 162 to 204 and library 2: amino acids 249 to 296, residues numbering referring to PDB#2J0O). Only solvent-exposed residues (exposed area > 10 Å^2^) within those regions were selected, with a theoretical diversity of each library of 1248 variants. After mutagenesis, PCR was performed to recover and amplify the library of *ipaD* single mutant genes. The final library was obtained by recombining mutant genes in the YSD plasmid pCT-L7.5.1 between NheI and BamHI restriction sites (kindly provided by K. Dane Wittrup, Addgene #42900).

### Yeast surface display

Preparation of competent yeast cells EBY100 (ATCC MYA-4941) and library transformation were performed according to Benatuil *et al* [68], with 2 μg of digested vector and a molar ratio of 25:1 (linear library/digested vector). Gap repair transformations were made in plasmid pCT-L7.5.1 between restriction sites BamHI and NheI. Precultures were performed by inoculating 250 mL of SD-CAA [6.7 g/L yeast nitrogen base without casamino acids, 20 g/L glucose, 5 g/L casamino acids, 100 mM sodium phosphate, pH 6.0] medium with 400 μL of transformed cells and incubated overnight at 30°C, 200 rpm. The saturated pre-culture (typically OD_600_ of 8–10) was passaged in order to obtain an initial culture OD_600_ of 0.25–0.50 in 50 mL. The culture was grown at 30°C until its OD_600_ reached 0.5–1.0. Cells were centrifuged and re-suspended in 50 mL of SG-CAA galactose induction medium [6.7 g/L yeast nitrogen base without casamino acids, 20 g/L galactose, 5 g/L casamino acids, 100 mM sodium phosphate, pH 6.0] and induced for 16–36 h at 20°C, 200 rpm.

### Fab preparation

Fab IpaD-318 was obtained by digestion of a previously described chimeric version of mAb IpaD-318 using the Pierce Fab Micro Preparation Kit [69].

### Flow cytometry

For library sorting, 10^8^ induced cells of each library were washed with 1 mL PBSF (PBS, BSA 0.1%) buffer. Cells were resuspended in 50 mL of a solution containing 160 pM of Fab IpaD-318 and 600 pM for VHH-20ipaD (FITC conjugate). These concentrations correspond to the experimental ½ K_D_ for both ligands determined on the surface of yeast cells as previously described [69]. Apparent binding affinity K_D app_ values for Fab IpaD-318 and VHH-20ipaD were 314 pM and 1.16 nM, respectively, in good accordance with previously reported values [28,69] (S4 Fig).

After incubation at 20°C, with agitation for 4 hours, cells were washed with 1 mL ice-cold PBSF to avoid dissociation. Cells were incubated on ice in the dark for 15 minutes with anti-human Ck antibody (Invitrogen #MA1-10385, APC conjugate), washed with 1 mL ice-cold PBSF and sorted with a BD FACS Aria III cytometer using BD FACSdiva software. Gates were defined to sort the top 5% of cells with decreased binding for mAb IpaD-318 and maintained binding for VHH-20ipaD. At least 100-fold of the theoretical diversity was sampled, and each gate was separately collected and recovered for 2 days in SD-CAA medium at 30°C.

### Deep sequencing and analysis of NGS data

Plasmid DNA of each yeast population was extracted and prepared for sequencing as described in Medina-Cucurella and Whitehead [70]. Two-step PCR was performed to amplify the region of interest and add Illumina adapters and barcodes for multiplexing (see sup data for primer sequences). Deep sequencing was performed with an Illumina MiSeq device (2x150 bp, v2 kit 300 cycles) with at least 250,000 reads per population. Reads were demultiplexed and each sample was processed separately using the Galaxy platform (https://usegalaxy.org/) using the functions described in Blankenberg *et al* [71]. First, paired reads were joined (Fastq Joiner). A trim was then performed (Fastq Trimmer) on reads to keep just the region of interest in the correct frame. A quality filter (Filter FASTQ) was applied to eliminate reads with a minimum quality score under 30. Next, DNA sequences were translated in protein sequences and identical sequences were grouped. Sequences not repeated at least two times were filtered out. Using the software RStudio, single-mutants were selected to allow calculation of enrichment ratios for each single mutation.

## Results

### Production and selection of anti-IpaD/SipD mAbs

Mice immunized with IpaD and presenting the highest polyclonal antibody titers specific for IpaD (S1A Fig) were chosen for fusion of spleen cells with myeloma cells according to the Köhler and Milstein method [72]. As the aim of this study was to obtain antibodies able to recognize homologous sequences of IpaD and SipD of *S*. *enterica* serovar Typhimurium and *S*. *flexneri 2a*, selection of antibodies was performed by double-screening ELISA, using IpaD and SipD biotinylated recombinant proteins (S1B Fig). These screenings led to a final selection of 20 mAbs able to cross-recognize IpaD and SipD (Table A in S1 Text).

In order to confirm that the selected mAbs were able to recognize the IpaD and SipD recombinant proteins, an analysis by immunoblot was performed. The immunoblot analysis showed that all tested mAbs recognized a band of approximately 37 kDa (theoretical molecular mass of the native recombinant proteins: 34 kDa for IpaD and 35.1 kDa for SipD) as well as other higher bands mainly for IpaD (around 75 kDa and higher), that we supposed to be either the protein in an oligomerized and not completely denatured or reduced form or aggregation in a non-native conformation induced by the denaturation (S2 Fig). It has to be noted that a cysteine residue present at the C-terminus of IpaD might be involved in a disulfide bond not completely reduced between two subunits and might explain the predominant form of the IpaD dimer [60]. These mAbs are all able to cross-recognize both IpaD and SipD proteins, confirming their selection on this criterion during the ELISA screening.

In order to select mAbs able to neutralize bacteria *in vivo*, the 20 mAbs recognizing both IpaD and SipD were first screened using 3 mice per antibody. They were injected twice (500 μg each injection) by the intraperitoneal route (IP) into BALB/c mice: first time, 24 h before intragastric infection with 100 LD50 of *S*. Typhimurium and, a second time, 4 days after the infection when the signs started to appear. Survival was monitored for 30 days and the number of mice surviving with each antibody is presented in Table B in S1 Text. Despite the small number of mice used, this first experiment allowed us to select 7 antibodies (IpaD-301, IpaD-304, IpaD-306, IpaD-311, IpaD-312, IpaD-317, IpaD-318) able to provide a protection, for further analysis.

### IpaD-301 and IpaD-318 mAbs conferred protection against *S*. *flexneri 2a* and *S*. Typhimurium challenge

**Fig 1:**
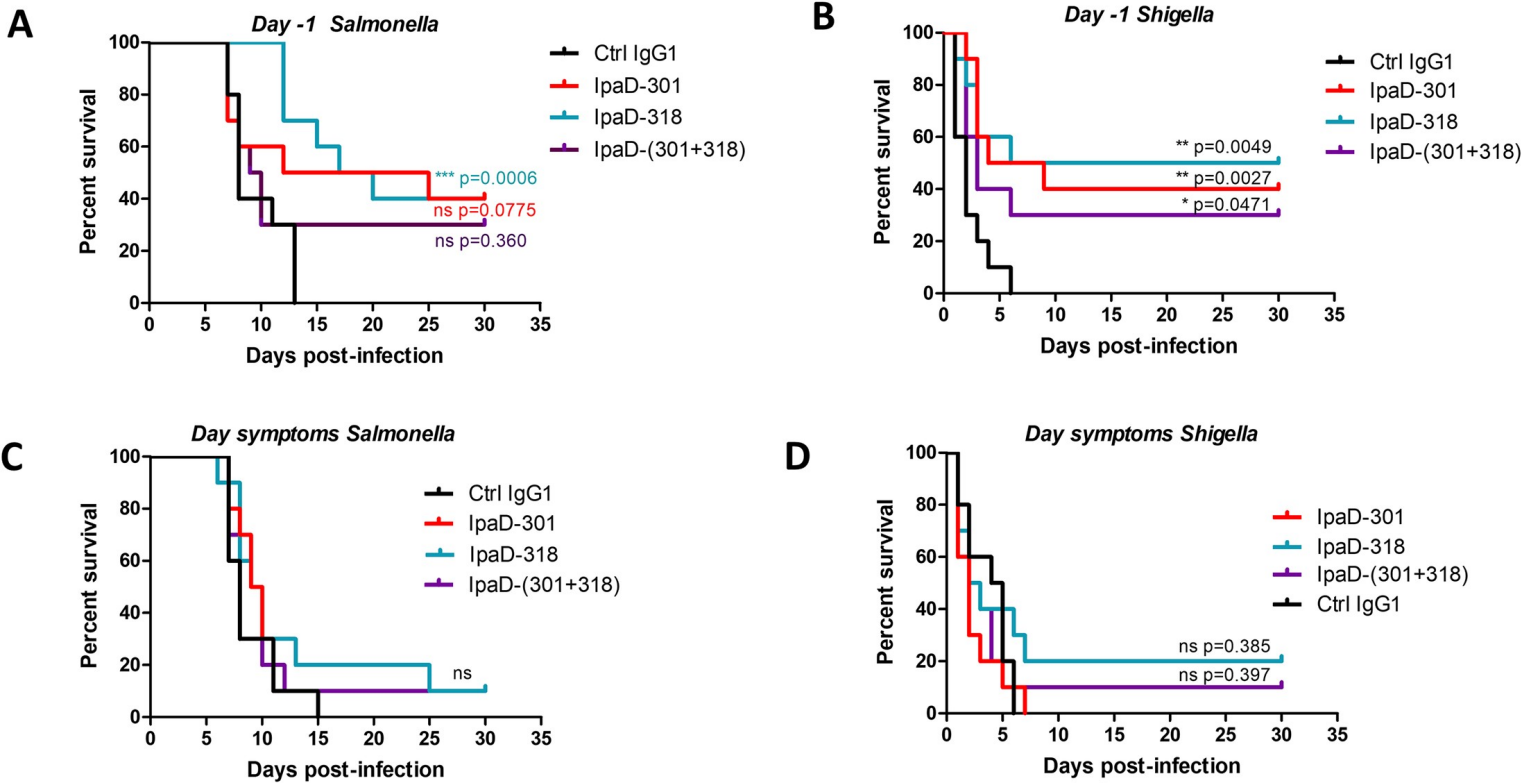
*In vivo* protective activity of anti-IpaD/SipD antibodies against *S*. *flexneri 2a* and S. Typhimurium challenges. Mice (N = 10) were injected with a total of 300 μg/mouse of the indicated anti-IpaD/SipD antibodies (alone or in combination) 24 h before (A, B) the intragastric (*S*. Typhimurium, A, C) or intranasal (*S*. *flexneri 2a*, B, D) 100 LD50 challenge and at the onset of the signs (C, D). Survival was monitored for 30 days. Statistical analysis was performed using a log-rank (Mantel-Cox) test comparing mice treated with mAbs versus control mice treated by a control non-relevant IgG1 antibody.

The seven selected antibodies were tested using ten mice per group with one intraperitoneal injection (300 μg per mouse) 24 h before challenge with 100 LD50 of *S*. Typhimurium (S3 Fig). The two best antibodies IpaD-301 and IpaD-318 mAbs provided 40% of protection (Fig 1A) and were selected for their ability to protect mice from infection with *S*. *flexneri 2a* using the same protocol as the one used for *S*. Typhimurium (Fig 1B). When antibodies were administered to mice 24 h before the 100 LD50 *S*. *flexneri 2a* intranasal challenge, IpaD-301 and IpaD-318 antibodies conferred a protection equivalent to the one observed for S. Typhimurium (40% and 50% protection, respectively (Fig 1B)). The antibody combination (IpaD-301 together with IpaD-318) did not increase the protective efficacy neither for *S*. Typhimurium nor for *S*. *flexneri 2a* (30% protection for both of them, Fig 1A and 1B). Moreover, the amino acid sequences of these two antibodies determined previously are very close (7 (none in CDRs) and 8 (4 in CDRs) mutations in the heavy chain and the light chain, respectively) [73] suggesting that those antibodies might share a common epitope on SipD/IpaD and would be mutually exclusive. We thus decided to subsequently focus on a more in-depth analysis of IpaD-318 only.

When mice were treated 5 days after *S*. Typhimurium challenge or 24 h after the *Shigella* challenge, corresponding respectively to the onset of the signs, the protection conferred by IpaD-318 dropped to a low and non-significant protection level of 10% and 20% respectively (Fig 1C and 1D).

Due to the greater efficacy of IpaD-318, we decided to go further in the analysis of its mechanism of action.

### mAb IpaD 318 increases contact mediated hemolysis and impairs HeLa cell invasion by *Shigella flexneri 2a*

**Fig 2:**
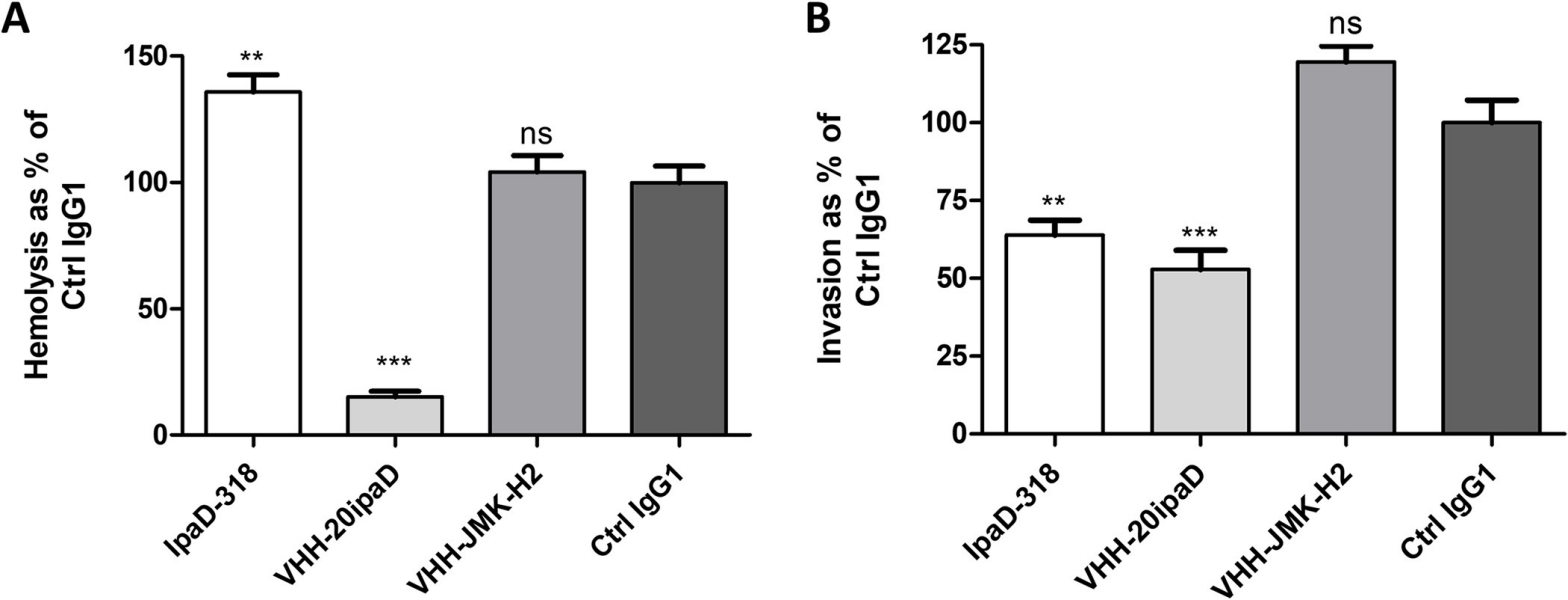
IpaD-318 *in vitro* protective capacity against *Shigella flexneri 2a*. **(A) Contact-mediated hemolysis**: the ability of antibodies to prevent hemoglobin release caused by contact-mediated lysis was evaluated and results are represented as % of control IgG1 (set at 100%). Data are representative of three independent experiments, mean+SEM values. (**B**) **HeLa cell invasion**: the ability of antibodies to prevent HeLa cell invasion was evaluated and results are represented as % of control IgG1 (set at 100%). Data are representative of four independent experiments, mean +SEM values. Statistical analysis between antibodies and control IgG1 was performed using one-way ANOVA with Tukey’s post-hoc test (** p<0.01, ***p<0.001).

To better understand the protective effect of IpaD-318 against *Salmonella/Shigella* infection, we performed *in vitro* experiments to evaluate its ability to inhibit the formation of pores in the host cell membrane (determined using a contact-mediated hemolysis assay, Fig 2A) and the *S*. *flexneri 2a* invasion of HeLa cells (Fig 2B). The results obtained here show that the IpaD-318 antibody does not prevent and even promotes the hemolysis, unlike VHH 20ipaD, which blocks it, and VHH JMK-H2, which has no effect, confirming for these antibodies the results obtained by Barta and colleagues [28]. In contrast, IpaD-318 partially inhibits invasion of HeLa cells by *S*. *flexneri 2a*, and similarly to VHH 20ipaD, in comparison with the isotypic control antibody (Ctrl IgG1) (Fig 2B). VHH JMK-H2 has no effect on either hemolysis or invasion. These experiments show that mAb IpaD-318 and VHH 20ipaD have different mechanisms of action, and that IpaD-318 makes it possible to decouple the role of IpaD in pore formation from its role in bacterial cell invasion. It is described in the literature that hemolysis requires the presence of IpaD [33], and in particular its N-terminal part [60], the C-terminal part being necessary for invasion and control of effector secretion [60].

### Epitope of mAb IpaD-318 is conserved among different *Salmonella* serovars and *Shigella* species

**Fig 3:**
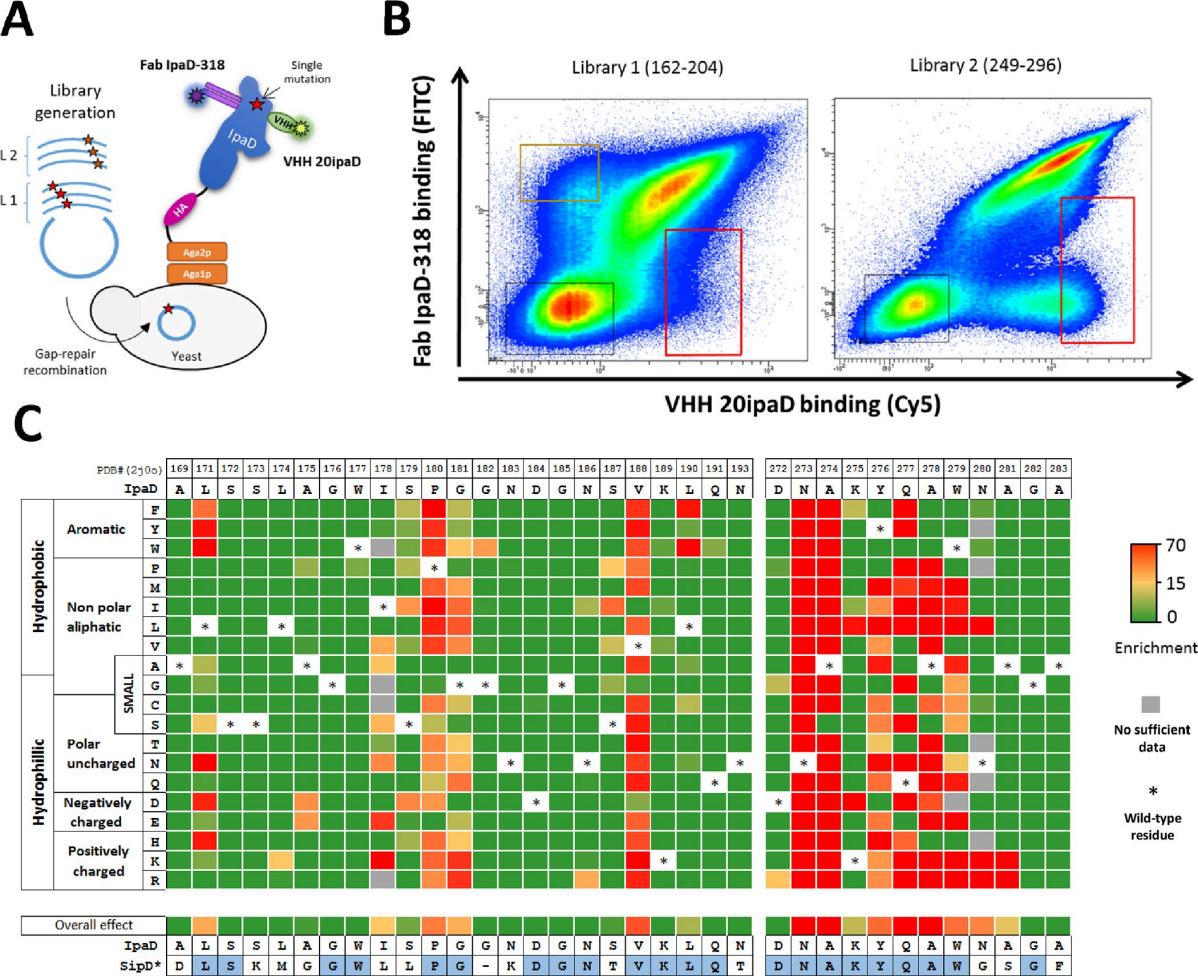
Epitope mapping of Fab IpaD-318 on the protein IpaD from *Shigella flexneri*. **(A)** General principle of functional screening by yeast surface display. A DNA library of single mutants of *ipaD* was transformed into yeast using gap repair recombination. Clones harboring IpaD mutations abolishing the binding of Fab IpaD-318 were sorted in FACS. To control the folding of the mutated IpaD antigen, a conformational VHH anti-IpaD compatible with Fab IpaD-318 was used (VHH 20ipaD, PDB #5VXM). **(B)** Bivariate flow cytometry analysis of libraries L1 and L2 of yeast cells expressing IpaD single amino acid variants on their surface. Each single dot on the plot designates the fluorescence intensity values for a single yeast cell corresponding to Fab IpaD-318 binding (FITC in y-axis) and VHH-20ipaD (Cy5 in x-axis) binding, respectively. Gates (red frames) were designed to sort yeast cells expressing IpaD mutants with altered affinity for Fab IpaD-318 and preserved affinity for VHH-20ipaD. **(C)** Heatmap representing enrichment values of each IpaD single mutant after functional sorting. For clarity, only the amino acids 169–193 and 272–283 are represented (see complete heatmap in S5A Fig). SipD*: structural alignment of SipD from *Salmonella enterica* Typhimurium (PDB #3NZZ) with IpaD (PDB #2J0O), identical residues are colored in blue. Overall effect corresponds to the mean of the enrichment values for all possible mutations at a selected position.

To gain insight into the mode of action of mAb IpaD-318 and in order to correlate it with the *in vitro* and *in vivo* experimental results, we sought to determine the localization of its epitope on IpaD/SipD using a yeast surface display (YSD) approach. IpaD was expressed on the surface of *S*. *cerevisiae* yeast cells in order to screen the ability of antibodies to bind the antigen (Fig 3A).

In a first experiment, we assessed the good folding of IpaD presented on the surface of yeast cells with two conformational VHHs (VHH-JMK-H2 and VHH-20ipaD) already published and for which epitopes on IpaD have been determined [28]. Both VHHs are conformational, recognizing discontinuous epitopes at the surface of the protein, and were able to recognize the protein at the surface of the yeasts, with high affinity (S4A Fig), attesting the proper folding of the protein. Moreover, competition and compatibility assays showed that mAb IpaD-318 competed with VHH-JMK-H2 but was compatible with VHH-20ipaD for its binding to wild-type IpaD (S4B Fig). Competition of mAb IpaD-318 with VHH-JMK-H2 suggests that these molecules might exhibit overlapping epitopes in the distal part of IpaD (opposite to the N- and C-terminus parts) [28,36].

To obtain high-resolution conformational epitope mapping, we used deep mutational scanning (DMS) associated with YSD to identify key IpaD amino acid positions necessary for IpaD-318 binding [74–79]. This method consists of the functional assessment of every possible amino acid change at each position in a protein. For DMS analysis, we selected residues located in the distal part of IpaD and accessible to solvent (exposed area > 10 Å^2^). As the distal part of IpaD is encoded by two separate regions in the amino acid sequence, two libraries were designed: library 1 (L1) from positions 162 to 204 (39 mutated residues) and library 2 (L2) from positions 249 to 296 (39 mutated residues). Site-saturation mutagenesis (SSM) libraries were generated following the “plasmid one pot saturation mutagenesis” method [67] to allow any of the 20 natural amino acids at the selected position using an NNS degenerate codon. DNA libraries were transformed into EBY100 yeast cells by gap repair recombination with the pCT-L751 expression plasmid (Fig 3A).

Libraries of yeast cells were induced to express the mutants on their surface before a functional screen in flow cytometry to identify clones harboring mutations abolishing Fab IpaD-318 binding. The conformational VHH-20ipaD, which is compatible with Fab IpaD-318, was used as a control to monitor the proper folding of mutants.

After labeling, analysis by fluorescence-activated cell sorting (FACS) revealed that the majority of cells expressing IpaD mutants had a strong fluorescence signal for both Fab IpaD-318 and VHH-20ipaD binding (Fig 3B). Variants with strong loss of Fab IpaD-318 binding were found in both libraries, although in different proportions, the vast majority of them belonging to library 2 encompassing residues 249 to 296. Conversely, a population exhibiting a loss of binding for VHH-20ipaD while maintaining binding for Fab IpaD-318 was observed in library 1 corresponding to residues 162 to 204, in accordance with the available crystallographic structure.

A gating strategy (in red, Fig 3B) was applied to select the fraction corresponding to the 5% of cells with the lowest Fab IpaD-318 binding and a preserved VHH-20ipaD binding. Cells with altered VHH-20ipaD were also selected in order to determine VHH-20ipaD’s epitope as a control (in orange, Fig 3B). Corresponding variants were sorted and cells were recovered in selective medium. Plasmid DNA of each library, before and after functional sorting, was extracted and prepared for deep sequencing on an Illumina MiSeq device (2*150bp) with at least 750,000 reads per population. For every mutant, enrichment ratios between unsorted and sorted libraries were calculated. Enrichment ratios were coded in color for each mutant and represented as a functionality heatmap (Fig 3C for partial Fab IpaD-318 heatmap and S5A Fig for complete Fab IpaD-318 heatmap, see S5B Fig for VHH-20ipaD heatmap). These ratios relative to the original binding interaction indicate whether a mutation causes a significant loss of binding (red), a moderate loss of binding (yellow) or no significant change in binding (green). To estimate the affinity reduction range of such mutations, two representative single mutants N273A and Y276T of IpaD were expressed at the surface of yeasts and their affinity constants were evaluated (S6 Fig). The “yellow” mutation Y276T causes a significant loss of affinity to Fab IpaD-318 as compared to wild-type IpaD (approximately 20 fold). The “red” N273A mutant is no longer recognized by Fab IpaD-318 even at the highest concentration tested (1μM), confirming a correlation between enrichment ratios and affinity.

The epitope of the control VHH-20ipaD determined by this method is represented on the published structure of the complex (S7 Fig). The epitope determined by DMS matches the epitope determined by crystallography, validating the method for identification of Fab IpaD-318’s epitope.

**Fig 4:**
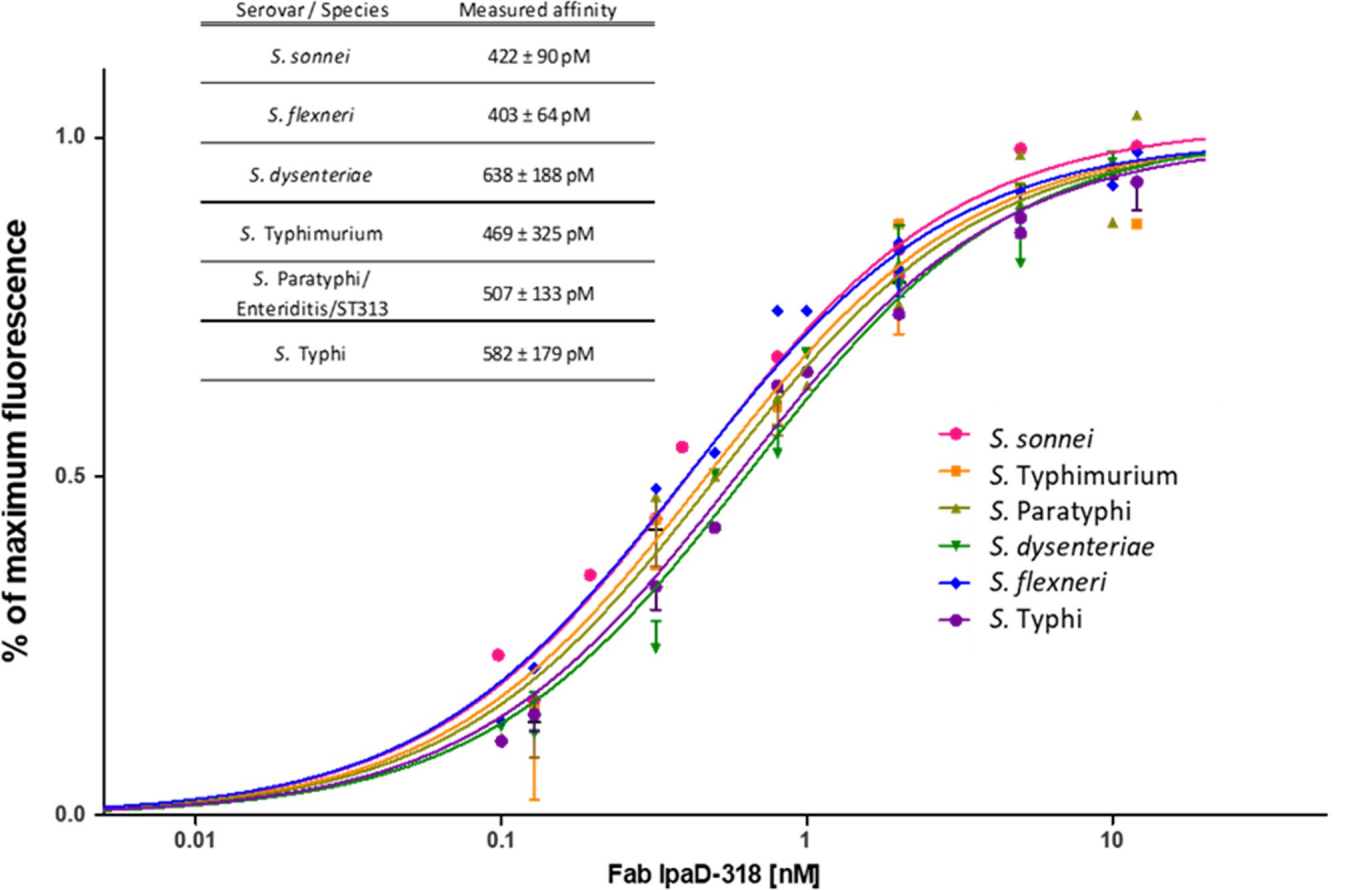
Binding affinity determination of Fab IpaD-318 on yeast cells displaying wild-type IpaD from several *Shigella*/*Salmonella enterica* species/serovars. IpaD or SipD from different species/serovars of *Salmonella* and *Shigella* was expressed at the surface of yeast cells. The fluorescence corresponding to Fab IpaD-318 binding was determined by FACS analysis in the presence of increasing concentrations of Fab. Measurements were done in duplicate with independent cultures and inductions. SipD from *S*. Paratyphi, *S*. Enteritidis and serovar ST313 all share the same sequence epitope. Apparent K_D_ values and 95% confidence intervals were determined using PRISM software with a one site-specific binding model.

When mutated, nine positions affect particularly the binding of Fab IpaD-318: P180, G181, V188, N273, A274, Y276, Q277, A278 and W279. Positions L171, I178, N280 and A281 also have a significant effect on the interaction when mutated. The alignment of the IpaD sequences of the main *Shigella* species (*flexneri*, *sonnei* and *dysenteriae*) as well as of SipD of the main *Salmonella* serovars (Typhi, Paratyphi, Typhimurium (including ST313) and Enteritidis) shows that the amino acids involved in the IpaD-318 epitope are all conserved in these different species/serovars (S8 Fig). In particular, eight of the nine main positions determined as the most important in DMS (P180, G181, N273, A274, Y276, Q277, A278 and W279) are strictly conserved. Only valine in position 188 is replaced by isoleucine in *S*. Enteritidis, Paratyphi, Typhi and Typhimurium ST313. According to DMS data, this replacement is the only one that has no impact on antibody binding, suggesting that Fab IpaD-318 should be able to recognize IpaD/SipD from all above-mentioned species/serovars. To confirm this hypothesis, IpaD/SipD from all these species/serovars were expressed at the surface of yeasts and evaluated for Fab IpaD-318 binding (Fig 4). The measured apparent K_D_ showed no difference between all species/serovars with values ranging from 403 pM to 638 pM.

**Fig 5:**
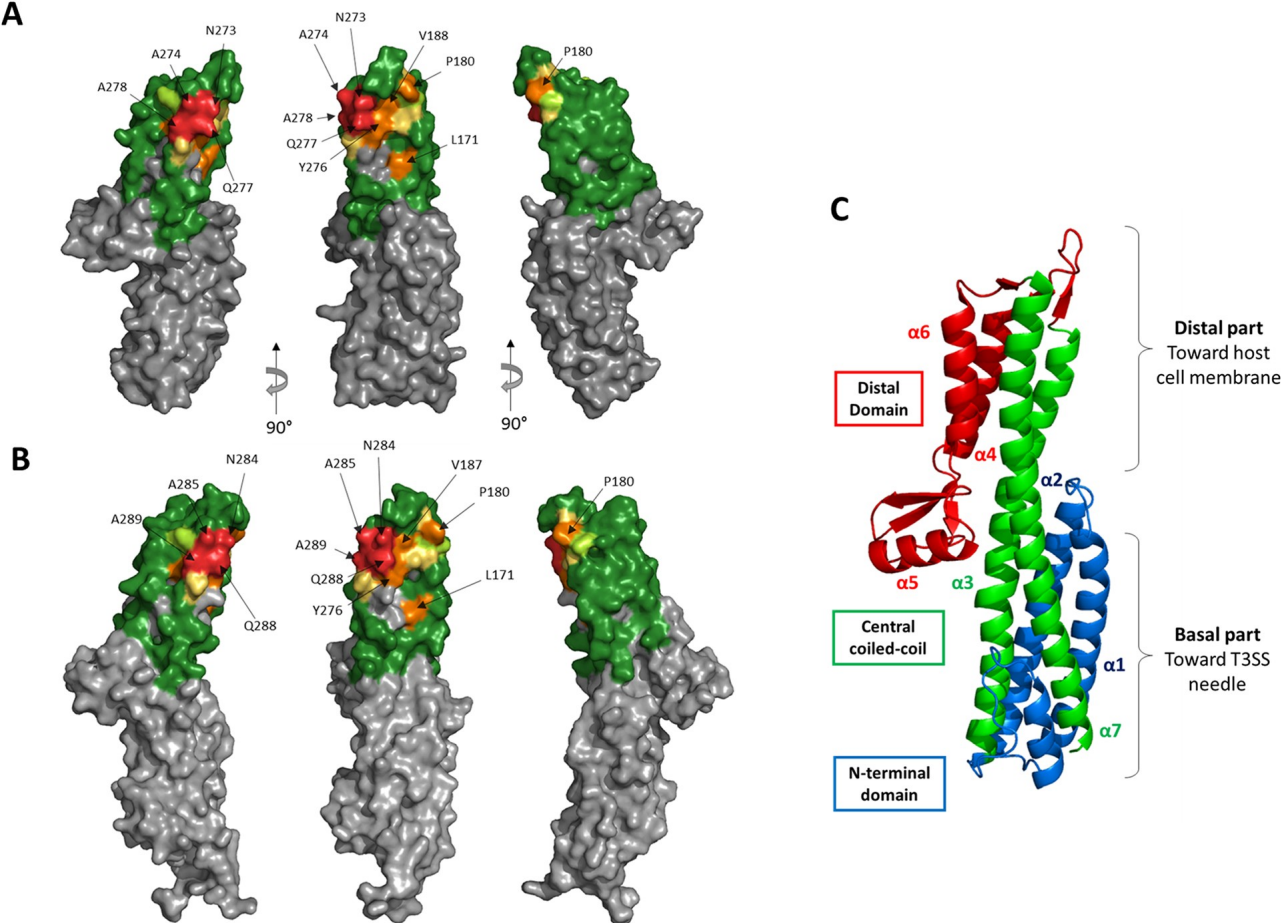
Structural representation of the epitope of mAb IpaD-318 on the antigen IpaD as determined by yeast display. **(A)** Residues were colored on the structure of IpaD (PDB #2J0O) by the overall effect color code of Fig 3C, representing the importance of each tested residue in the interaction. Grey residues were not tested. **(B)** Epitope representation of mAb IpaD-318 on the antigen SipD (PDB #3NZZ). **(C)** Structural description of IpaD. Domain segmentation of the protein as described in [28].

The average mutation effect for each position was determined and represented on IpaD structure (Fig 5A). The epitope of IpaD-318 determined by YSD is located in the distal part of IpaD, on the concave side of the protein. Key amino acids represented on the structure belong mainly to the apical end of the two central coiled-coil helices (residues 273–278) and to the apical loop between positions 175 and 191. These results are in good agreement with the epitope determined for the VHH-JMK-H2 that competes with Fab IpaD-318 for IpaD binding [28].

## Discussion

We have recently shown that SipD and IpaD were able to elicit a cross-protective effect against *Shigella* and *Salmonella* infections [21]. This study gave the first evidence of the interest of these proteins as potential targets to protect broadly against *Salmonella* and *Shigella* infection in development of new vaccines. To go deeper into the importance of the humoral response against conserved regions of the SipD and IpaD proteins and to provide evidence of its broad protective role, we decided to produce mAbs directed against conserved epitopes of both proteins and to evaluate their cross-protective effect *in vivo*. We selected the mAbs based on their ability to bind both proteins indifferently, on a common epitope at their surface. The most efficient antibodies IpaD-301 and IpaD-318 provided good protection against bacterial infections when administered intraperitoneally to mice one day before challenge by *S*. Typhimurium or *S*. *flexneri* (40 and 50%, respectively), showing that mAbs directed to shared epitopes of SipD/IpaD are sufficient to induce good protection against high challenges of *Salmonella* and *Shigella*. Peptide sequence proximity between IpaD-301 and IpaD-318 as well as competition assays on both antigens (SipD and IpaD) led us to consider that it was the same antibody and we used only IpaD-318 for the rest of the study [69]. These results confirm the important role of humoral response in the cross-protection observed after immunization by either of the two proteins against infection by either of these pathogens and IpaD-318 proved that a monocolonal antibody directed against specific and conserved regions of needle-tip proteins alone is essential to provide good cross-protection against high doses of *Salmonella* and *Shigella*. Although it is recognized that the mouse model of *Shigella* pulmonary infection is not ideal to mimic an intestinal infection, it is currently the one used by scientific community for the evaluation of vaccines in development. Protection fell dramatically when the antibody was administered at the onset of the symptoms (24 h or 5 days after the challenge by *Shigella* and *Salmonella*, respectively), suggesting that multiplication of the bacteria was too great given the very high challenge dose and/or their accessibility to antibodies was impaired. Indeed, *Shigella* and *Salmonella* bacteria replicate intracellularly within the host cells. Once they have entered the cell, the bacteria can no longer be reached by the antibodies, which partly explains the lack of effectiveness of treatment at a late stage of the disease.

To our knowledge, this is the first description of mAbs able to provide cross-protection *in vivo* against a high challenge of *Salmonella* and *Shigella* infections and directed against shared epitopes of IpaD and SipD T3SS needle-tip proteins. For this reason, we decided to determine the binding epitope of mAb IpaD-318 on IpaD with the aim of understanding how this anti-IpaD/SipD antibody protects against the virulence of *Salmonella/Shigella* pathogens.

The epitope of mAb IpaD-318 is composed of discontinuous amino acids in the peptide sequence, leading to a conformational epitope that is highly conserved among lots of serovars/species of *Salmonella* and *Shigella*, as shown in the sequence alignments for *S*. *sonnei*, *flexneri*, *dysenteriae* and *S*. Typhimurium, Enteritidis, Paratyphi and Typhi, and as confirmed by the binding experiments and K_D_ measurements that are very similar for the different IpaD and SipD of these bacteria. These results allow us to be confident about the ability of this antibody to protect against the serotypes not tested in this study. The epitope is located in the distal part of IpaD/SipD, which is the side of the tip protein directed towards the host membrane. *In vitro* experiments on the impact of IpaD-318 on pore formation (contact mediated hemolysis) and cell invasion by *Shigella* unexpectedly and interestingly showed that this antibody had the ability to dissociate the two functions of IpaD. Binding of the antibody promotes hemolysis, suggesting that the antibody does not inhibit oligomerisation of IpaD, but would prevent it from acting as a controller of IpaB/IpaC effector secretion. It has been described that mutants not expressing IpaD lose the capacity for hemolysis but constitutively secrete the IpaB and IpaC translocons [33]. The phenotype observed here in presence of IpaD-318 is intermediate, suggesting the presence of IpaD on the needle tip (since hemolysis occurs) but in a state/conformation that increases pore formation with a partial loss of control of translocon protein secretion. The fact that IpaD-318 inhibits the invasion of HeLa cells by bacteria tends to confirm that the tip is not in an active and/or stable conformation and that, although allowing pore formation when contacts between bacteria and cells are forced artificially by centrifugation in the hemolysis experiment, the anchoring of the bacteria to the cell in a more physiological environment would be too labile and not sufficient to allow the initiation of secretion of late effectors ultimately leading to invasion of the cell by the bacteria. These results are in agreement with the determined epitope of mAb IpaD-318, represented in S9 Fig on the complex proposed by Blocker and co-workers, which pleads in favour of its association not after the complete formation of the pentamer, because in this configuration the epitope does not seem accessible to the antibody, but rather at an earlier stage, probably by preventing the positioning of the fifth IpaD subunit and its subsequent replacement by IpaB to form the active functional tip complex (according to the model described in [44]) and the docking to the cell by the transmembrane domain of IpaB. This insertion, which would stabilize the anchoring of the bacterium to the cell, and would then allow a signal to trigger the secretion of late effectors towards the base of the needle, would therefore be prevented by the binding of mAb IpaD-318 instead of the 5th partner. Our experimental results and this hypothesis are in agreement with the model proposed by Johnson *et al* (2007) who showed that the C-terminal domain of IpaD was more involved in its binding with IpaB than with itself and that a weak assembly of IpaD with IpaB [22] or even the absence of IpaB [80] led to leakage of the effectors through the opening of the canal and uncontrolled constitutive secretion. These results are also in agreement with the characterization of mutants in the C-terminal part of IpaD that modify the invasion function and the control of IpaB/IpaC secretion. Schiavolin *et al*, identified a region (aa 271–280) which, when deleted, altered IpaB surface exposition, and induces secretion of early effectors [81]. More precisely, Meghraoui *et al*, mutated a single amino acid (Y276A) which results in the increased secretion of effectors and cellular invasion and would maintain IpaD in an active conformational state, close to that occurring in the intestinal lumen [82]. On the contrary, Roehrich and coworkers found point mutations in IpaD that led to impairment of effector secretion induction and invasion [83]. Among the 4 most affected mutants, 3 are located at the top of the TC: N186, N273 and Q277. N273 and Q277 belong to and N186 borders the IpaD-318 epitope. These residues could be involved in a structural rearrangement of subunits at the interface with IpaB, leading us to hypothesize that the mAb IpaD-318 might prevent the binding of IpaB to IpaD and thus prevent the necessary structural rearrangement needed for T3SS activation, host cell sensing and effector secretion. Recently, Barta *et al* generated and characterized four anti-IpaD VHHs [28]. They evaluated the potential of *Shigella*-mediated hemolysis inhibition of their VHHs *in vitro* and also solved their structure in complex with IpaD. The epitope of one of the VHHs tested (JMK-H2) overlaps but is not identical to that of mAb IpaD-318. Surprisingly and in contrast to IpaD-318, which is protective against both *Salmonella* and *Shigella* infections *in vivo* in a murine model through inhibition of cell invasion, JMK-H2 was not able to inhibit *Shigella*-mediated hemolysis or cell invasion in *in vitro* experiments.

Given that SipD and IpaD share the same epitope and because of their equivalent protection by the mAb IpaD-318, it should be underlined that this antibody also helps to provide new insights into the role and mechanism of action of SipD in host cell infection by *Salmonella*, which is much less described than that of *Shigella* and which is confirmed to be very similar to that of *Shigella*. Although SipD (like IpaD) has been proved to be essential for host cell invasion and that its deletion led to increased secretion of effector proteins [84], a heteropentamer of SipB/SipD at the tip of T3SS has never been described for *Salmonella* and results obtained in this study could help to gain further insights into the molecular organization of the Salmonella tip complex. Interestingly, it has already been demonstrated that IpaB interacts with SipD in certain conditions [36].

In conclusion, despite relatively weak identity sequence (around 40%), we have been able to obtain an antibody directed against conserved sequences of needle-tip proteins of T3SS which on its own confers cross-genus protection against *Salmonella* and *Shigella* infections. Considering its epitope, the sequence alignment and binding experiments, this antibody should help to provide protection against most *Salmonella* serovars (*S*. Typhi, *S*. Typhimurium, *S*. Enteriditis, *S*. Paratyphi, and ST313 clone of *S*. Typhimurium known to be responsible for invasive nontyphoidal salmonellosis in sub-Saharan Africa) and *Shigella* species (*S*. *flexneri*, *S*. *dysenteriae*, *S*. *sonnei*) known to be pathogenic in humans. Although the antibody only works prophylactically, it is the first described in the literature that can at least protect partially against both *Shigella* and *Salmonella* infections *in vivo* in mice. Since the region targeted by this mAb is essential to the pathogen’s virulence and is conserved among *Salmonella* and *Shigella* bacteria, it would be of interest to consider the development and evaluation of either chemical compounds or vaccines targeting this specific area for broad-spectrum activity. Moreover considering recent advances in recombinant mAb engineering and studies done for oral administration of mAbs in particular for *Salmonella* infection [85], combatting diarrheal diseases induced by *Salmonella* and *Shigella* through administration of mAb alone or in combination with other molecules might become realistic and cost-effective [86] in different scenarios like: i) in the event of an epidemic (in order to protect people not yet ill but at risk), ii) in prevention for at risk travelers or the military personnel before their intervention on the scene of military operations iii) in order to limit the gravity of the symptoms and iv) in case of antibiotic resistant strains for which an antibiotic treatment combined with passive immunotherapy might be of interest and might help to reduce minimal inhibitory concentrations of antibiotics by a synergistic effect and thus limit the concentration of antibiotics used while allowing it to be active.

## Supporting information

S1 Fig Polyclonal antibody titers of IpaD-immunized BALB/c mice. **A- Evaluation of immune polyclonal response by enzyme immunoassay.** The immune polyclonal response was evaluated by enzyme immunoassay (EIA) with serial dilutions of plasma (P) harvested at different times during immunization by IpaD recombinant protein. These two mice presenting the highest immune response were selected for selection of mAbs. **B. Principle of the Enzyme Immunoassay**. Culture supernatants for the final selection of mAbs able to recognize both SipD and IpaD were screened using differential sandwich ELISA. Each culture supernatant containing mAbs of interest was tested using either biotinylated IpaD or SipD. The same ELISA test was performed to measure the concentrations of circulating antibodies (immune response after immunizations) using recombinant biotinylated IpaD.
(TIF)

S2 Fig Recognition of IpaD (top) and SipD (bottom) recombinant T3SS proteins with anti-IpaD/SipD monoclonal antibodies. Western blotting was performed with 10 ng/well (first well) and 100 ng/well (second well) of IpaD or SipD against 18 purified anti-IpaD/SipD mAbs (4 μg/mL). The numbers of mAbs are indicated below each immunoblot. Numbers on the left indicate the molecular weight markers in kDa.
(TIF)

S3 Fig *In vivo* protective mAb screening. Survival curves of mice treated with monoclonal antibodies (10 mice per antibody) 14 h before infection with 100 LD50 of *S*. Typhimurium. P-value was calculated in comparison with the control with the log-rank (Mantel-Cox) test.
(TIF)

S4 Fig Binding capability and compatibility assay of VHH-JMK-H2 and VHH-20ipaD vs mAb IpaD-318. **A- Apparent K**_**D**_ **determination of Fab IpaD-318 and VHH-20ipaD on yeast cells expressing wild-type IpaD**. Wild-type IpaD was expressed on the surface of yeast cells. The fluorescence corresponding to Fab or VHH binding was determined by FACS analysis in the presence of increasing concentrations of the corresponding antibody fragment. Apparent K_D_ values and 95% confidence intervals were determined using PRISM software with a one site-specific binding model. Apparent K_D_ values were measured at 350 ± 70 pM for Fab IpaD-318 and 1.15 ± 0.30 nM for VHH-20ipaD. **B- Binding competition and compatibility assay of VHH-JMK-H2 and VHH-20ipaD vs mAb IpaD-318.** Wild-type IpaD was expressed on the surface of *S*. *cerevisiae* cells EBY100 to determine VHH-JMK-H2 and 20ipaD competed with mAb IpaD-318 for IpaD binding. The binding fluorescence of biotinylated mAb IpaD-318 on the surface of yeasts is shown as histograms. Prior to mAb IpaD-318 binding, PBS (control), VHH-20ipaD (1 μM) or VHH-JMK-H2 (1 μM) was added to the yeast cells expressing IpaD. Cells were washed and incubated with 1 nM biotinylated mAb IpaD-318. Binding fluorescence (SA-PE) was monitored by flow cytometry.
(TIF)

S5 Fig **Complete heatmap representing enrichment values of each IpaD mutant after sorting of variants with altered Fab IpaD-318 binding (A) or VHH-20ipaD binding (B).** For a mutation, a strong enrichment value represents a loss of binding. Only exposed residues (bold letters) were mutated.
(TIF)

S6 Fig Binding affinity determination of Fab IpaD-318 on yeast cells displaying IpaD mutants. Single IpaD mutants N273A and Y276T (respectively classified as red and yellow mutations in Fig 3C heatmap) along with wild-type IpaD (*S*. *flexneri*) were expressed at the surface of yeast cells. The fluorescence corresponding to Fab IpaD-318 binding was determined by FACS analysis in the presence of increasing concentrations of Fab. Measurements were done in duplicates. Apparent K_D_ values and 95% confidence intervals were determined using PRISM software with a one site-specific binding model.
(TIF)

S7 Fig Structural representation of the epitope of VHH-20ipaD on the antigen IpaD as determined by yeast display on the structure of the complex. Residues were colored on the structure of the complex IpaD/VHH-20ipaD (PDB #5VXM) by the overall effect color code of Figs 3C and S5B, representing the importance of each tested residue in the interaction. Grey residues were not tested.
(TIF)

S8 Fig Multiple alignment of IpaD and SipD sequences from different serovars / species. Multiple alignment of IpaD from three *Shigella* species (*S*. *flexneri*, *S*. *sonnei* and *S*. *dysenteriae*) and SipD from five *Salmonella enterica* serovars (Enteritidis, Paratyphi, Typhimurium, Typhi and Typhimurium ST313). *S*. Enteritidis, *S*. Paratyphi and *S*. Typhimurium ST313 all share the same SipD sequence epitope. *In vivo* protection experiments were performed using *Shigella flexneri 2a* and *Salmonella* Typhimurium. Clustal omega was used to perform the alignments (www.ebi.ac.uk/Tools/msa/clustalo/). Red framing refers to key positions for Fab IpaD-318 binding as determined by deep mutational scanning experiments.
(TIF)

S9 Fig Representation of the epitope of mAb IpaD-318 on the proposed model for the tip complex. Representation of the epitope of mAb IpaD-318 on the proposed model (PDB #4d3e) for the tip complex of Blocker and co-workers. In this model, the fifth member of the pentamer is supposed to be IpaB (not represented). (A) Side view. (B) Upper View. (C) Side view focused on the epitope lacking the 4^th^ subunit.
(TIF)

S1 Text **Table A. Numbers and isotypes of IpaD/SipD monoclonal antibodies produced against IpaD protein.** The isotypes were identified by a sandwich ELISA test using antibodies specific to each isotype. Although recognizing both proteins and as they were obtained from mice immunized with IpaD, they were named “IpaD”. **Table B. *In vivo* selection of anti-IpaD/SipD monoclonal antibodies with neutralizing activity against intranasal *S*. *flexneri 2a* or intragastric *S*. Typhimurium challenge.** Mice (N = 3) were injected twice with 500 μg of each mAb (24 hours before the challenge and 4 days later) by the intraperitoneal route and were challenged with 100 LD50 of *S*. Typhimurium by the intragastric route. Survival was monitored for 30 days.
(DOCX)
