# Supplementary figures and images for: An antibody targeting type III secretion system induces broad protection against Salmonella and Shigella infections
Source: PLoS Negl Trop Dis. 2021 Mar 12;15(3):e0009231. doi: 10.1371/journal.pntd.0009231 (PMC7990167; doi:10.1371/journal.pntd.0009231)

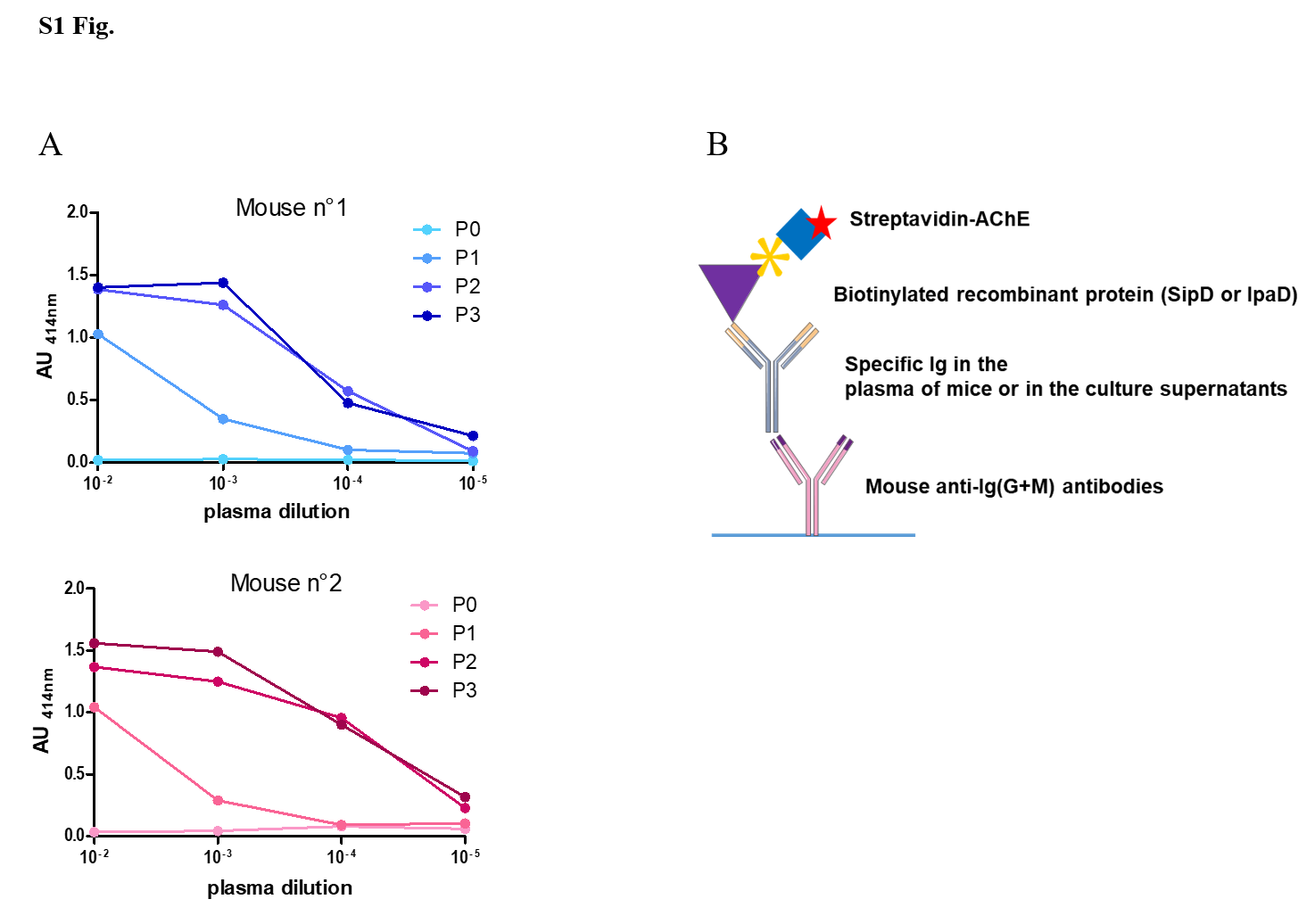

Supplement: S1 Fig — A- Evaluation of immune polyclonal response by enzyme immunoassay. The immune polyclonal response was evaluated by enzyme immunoassay (EIA) with serial dilutions of plasma (P) harvested at different times during immunization by IpaD recombinant protein. These two mice presenting the highest immune response were selected for selection of mAbs. B. Principle of the Enzyme Immunoassay. Culture supernatants for the final selection of mAbs able to recognize both SipD and IpaD were screened using differential sandwich ELISA. Each culture supernatant containing mAbs of interest was tested using either biotinylated IpaD or SipD. The same ELISA test was performed to measure the concentrations of circulating antibodies (immune response after immunizations) using recombinant biotinylated IpaD. (TIF) [file pntd.0009231.s001.tif]

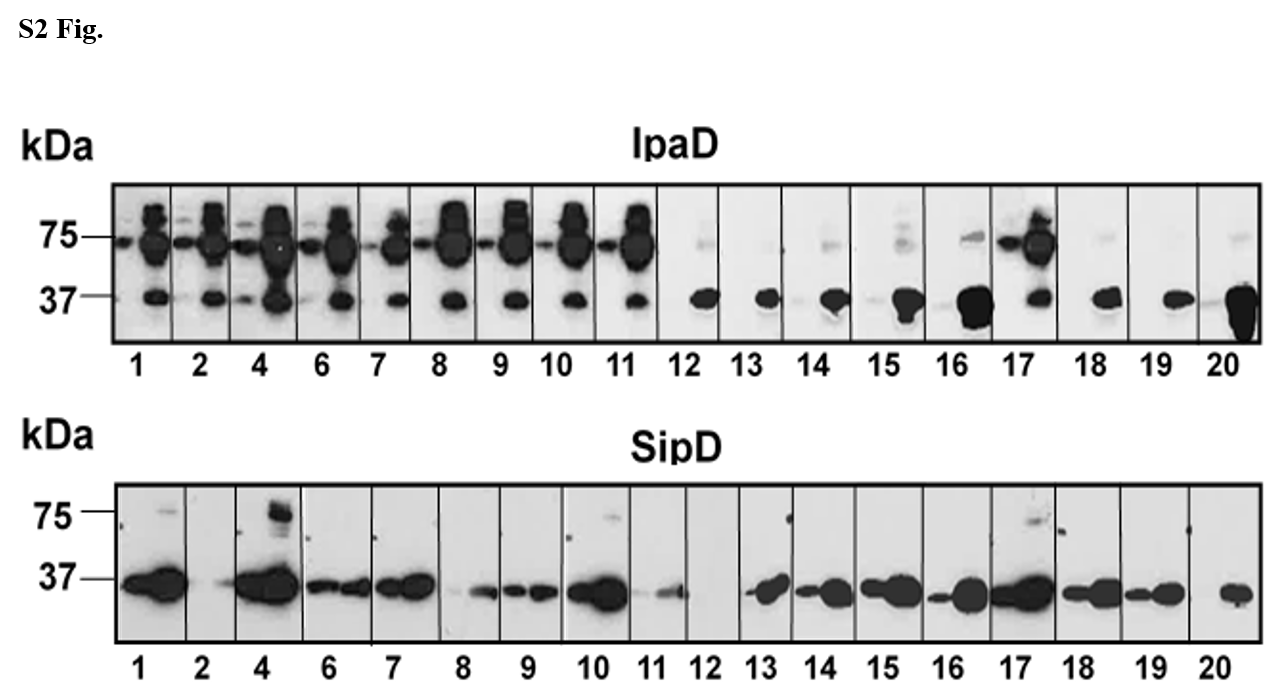

Supplement: S2 Fig — Western blotting was performed with 10 ng/well (first well) and 100 ng/well (second well) of IpaD or SipD against 18 purified anti-IpaD/SipD mAbs (4 μg/mL). The numbers of mAbs are indicated below each immunoblot. Numbers on the left indicate the molecular weight markers in kDa. (TIF) [file pntd.0009231.s002.tif]

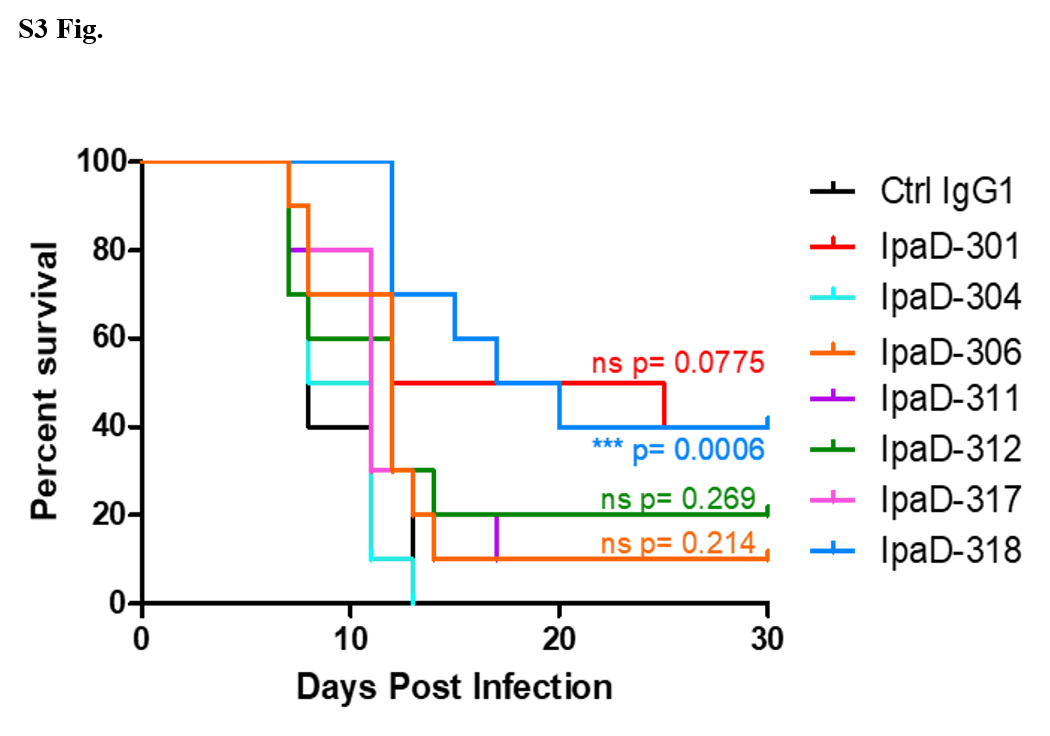

Supplement: S3 Fig — Survival curves of mice treated with monoclonal antibodies (10 mice per antibody) 14 h before infection with 100 LD50 of S. Typhimurium. P-value was calculated in comparison with the control with the log-rank (Mantel-Cox) test. (TIF) [file pntd.0009231.s003.tif]

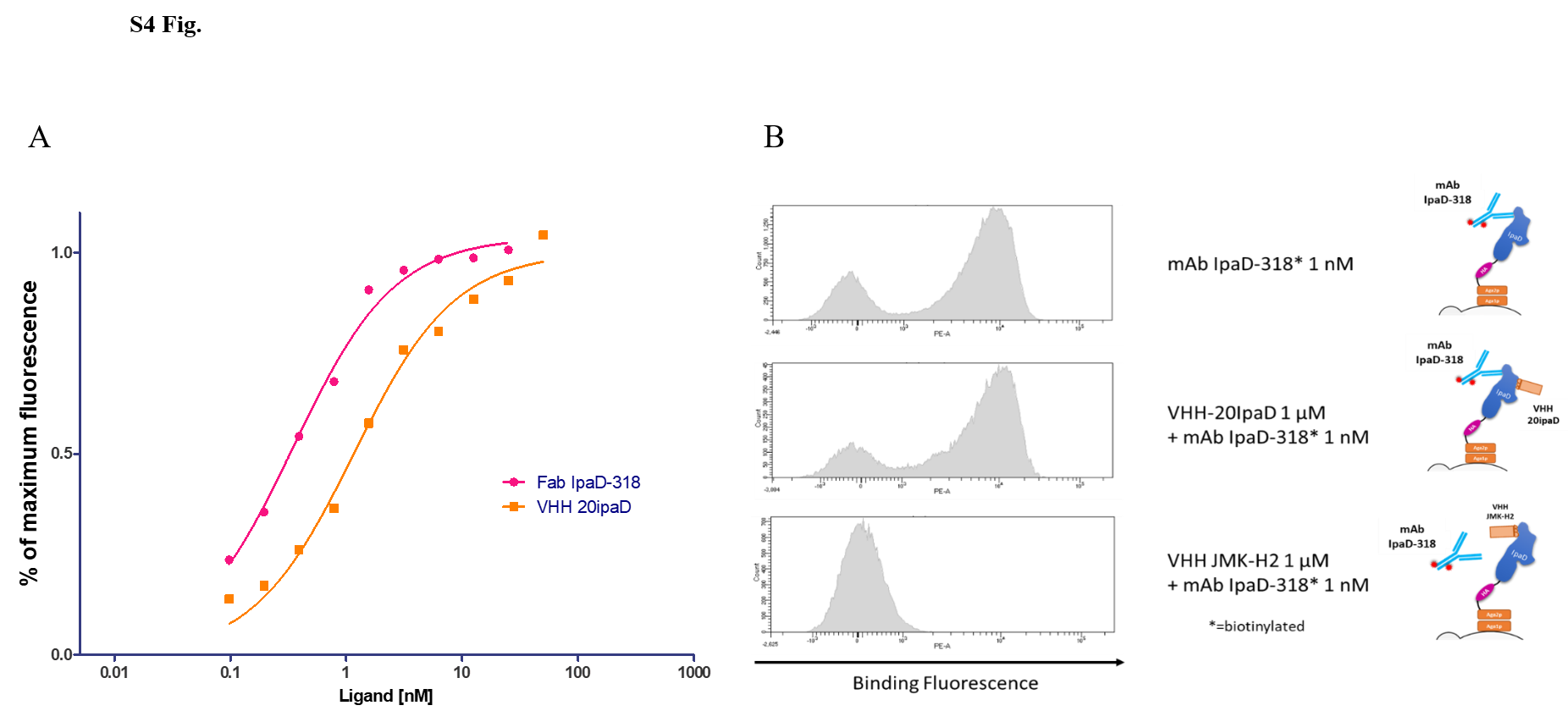

Supplement: S4 Fig — A- Apparent KD determination of Fab IpaD-318 and VHH-20ipaD on yeast cells expressing wild-type IpaD. Wild-type IpaD was expressed on the surface of yeast cells. The fluorescence corresponding to Fab or VHH binding was determined by FACS analysis in the presence of increasing concentrations of the corresponding antibody fragment. Apparent KD values and 95% confidence intervals were determined using PRISM software with a one site-specific binding model. Apparent KD values were measured at 350 ± 70 pM for Fab IpaD-318 and 1.15 ± 0.30 nM for VHH-20ipaD. B- Binding competition and compatibility assay of VHH-JMK-H2 and VHH-20ipaD vs mAb IpaD-318. Wild-type IpaD was expressed on the surface of S. cerevisiae cells EBY100 to determine VHH-JMK-H2 and 20ipaD competed with mAb IpaD-318 for IpaD binding. The binding fluorescence of biotinylated mAb IpaD-318 on the surface of yeasts is shown as histograms. Prior to mAb IpaD-318 binding, PBS (control), VHH-20ipaD (1 μM) or VHH-JMK-H2 (1 μM) was added to the yeast cells expressing IpaD. Cells were washed and incubated with 1 nM biotinylated mAb IpaD-318. Binding fluorescence (SA-PE) was monitored by flow cytometry. (TIF) [file pntd.0009231.s004.tif]

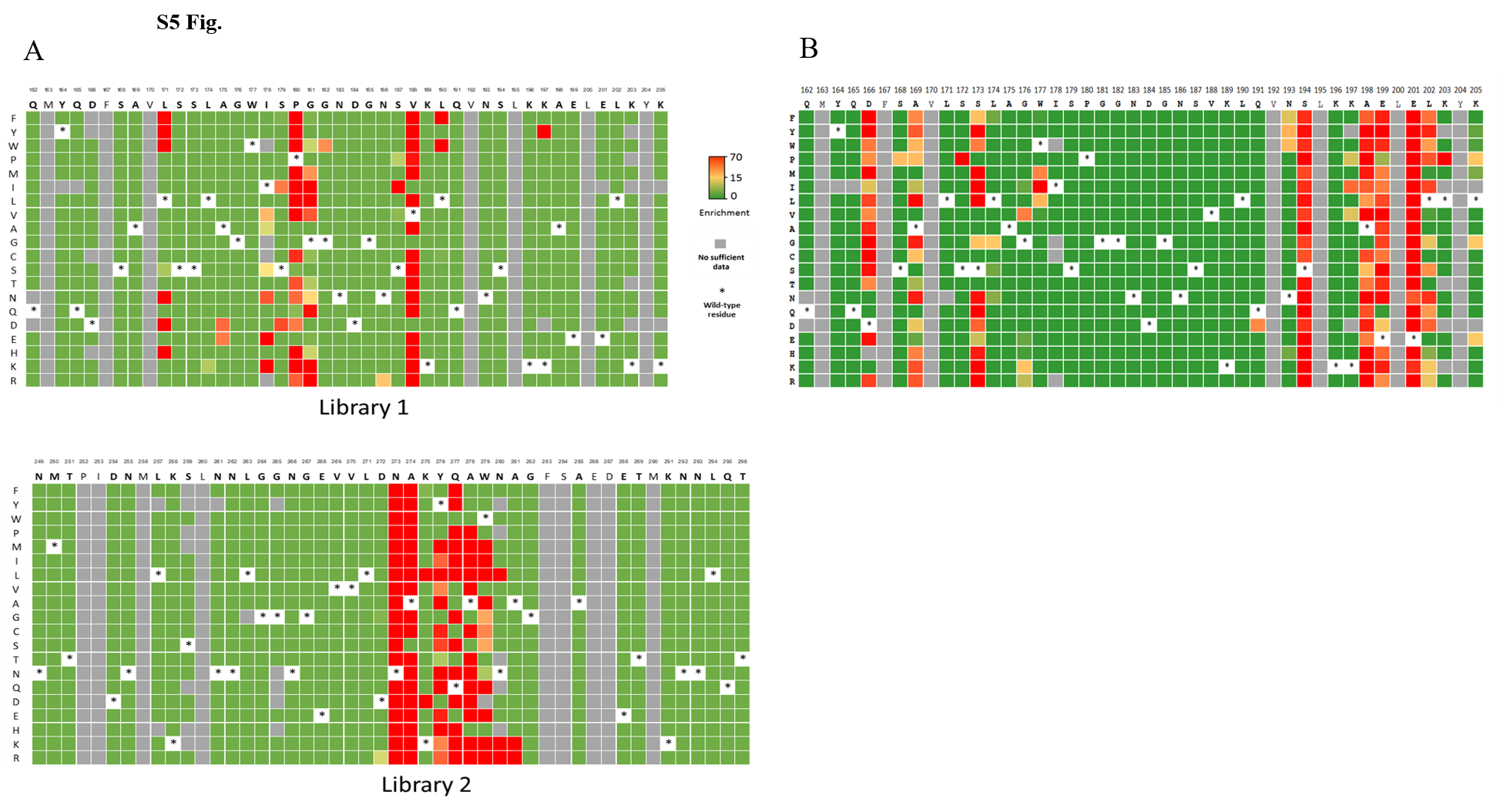

Supplement: S5 Fig — Complete heatmap representing enrichment values of each IpaD mutant after sorting of variants with altered Fab IpaD-318 binding (A) or VHH-20ipaD binding (B). For a mutation, a strong enrichment value represents a loss of binding. Only exposed residues (bold letters) were mutated. (TIF) [file pntd.0009231.s005.tif]

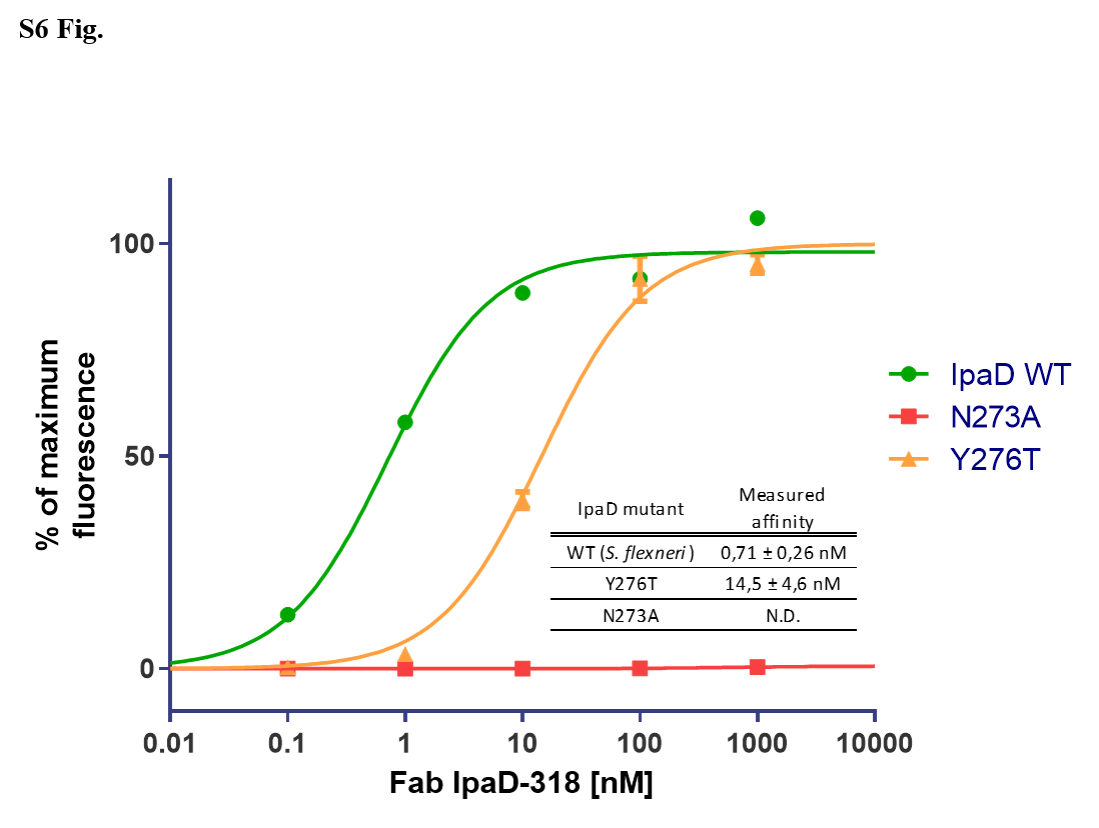

Supplement: S6 Fig — Single IpaD mutants N273A and Y276T (respectively classified as red and yellow mutations in Fig 3C heatmap) along with wild-type IpaD (S. flexneri) were expressed at the surface of yeast cells. The fluorescence corresponding to Fab IpaD-318 binding was determined by FACS analysis in the presence of increasing concentrations of Fab. Measurements were done in duplicates. Apparent KD values and 95% confidence intervals were determined using PRISM software with a one site-specific binding model. (TIF) [file pntd.0009231.s006.tif]

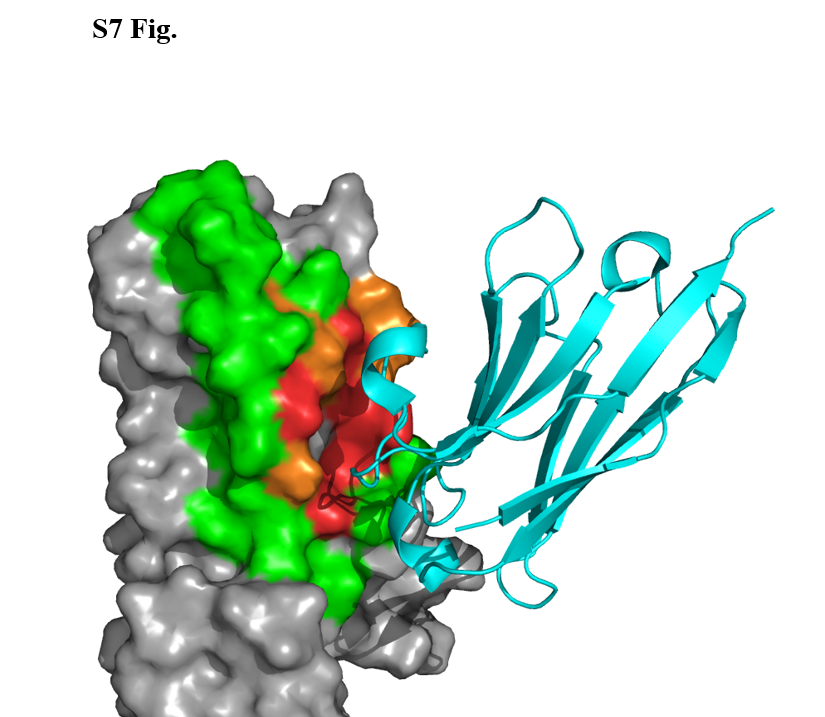

Supplement: S7 Fig — Residues were colored on the structure of the complex IpaD/VHH-20ipaD (PDB #5VXM) by the overall effect color code of Figs 3C and S5B, representing the importance of each tested residue in the interaction. Grey residues were not tested. (TIF) [file pntd.0009231.s007.tif]

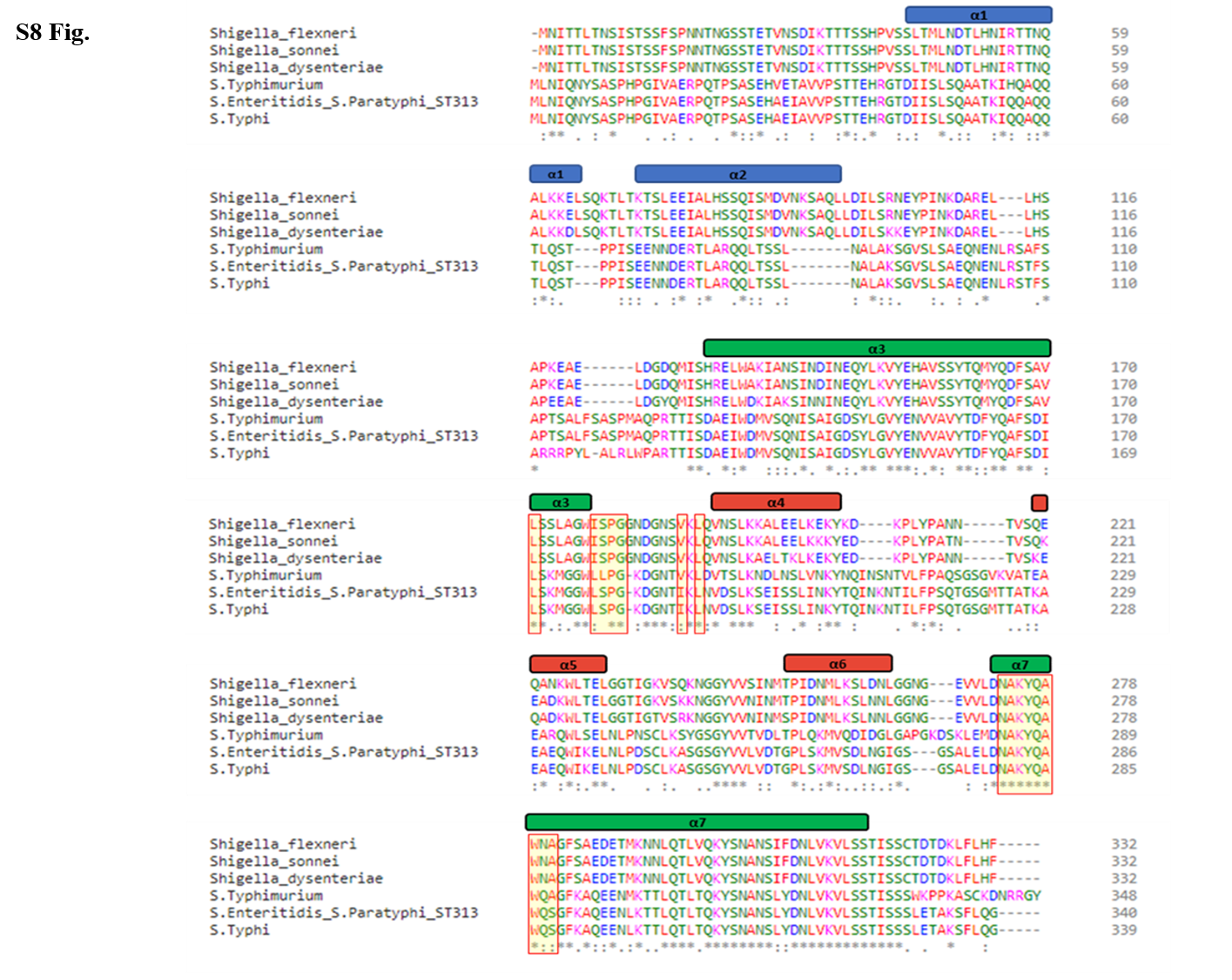

Supplement: S8 Fig — Multiple alignment of IpaD from three Shigella species (S. flexneri, S. sonnei and S. dysenteriae) and SipD from five Salmonella enterica serovars (Enteritidis, Paratyphi, Typhimurium, Typhi and Typhimurium ST313). S. Enteritidis, S. Paratyphi and S. Typhimurium ST313 all share the same SipD sequence epitope. In vivo protection experiments were performed using Shigella flexneri 2a and Salmonella Typhimurium. Clustal omega was used to perform the alignments (www.ebi.ac.uk/Tools/msa/clustalo/). Red framing refers to key positions for Fab IpaD-318 binding as determined by deep mutational scanning experiments. (TIF) [file pntd.0009231.s008.tif]

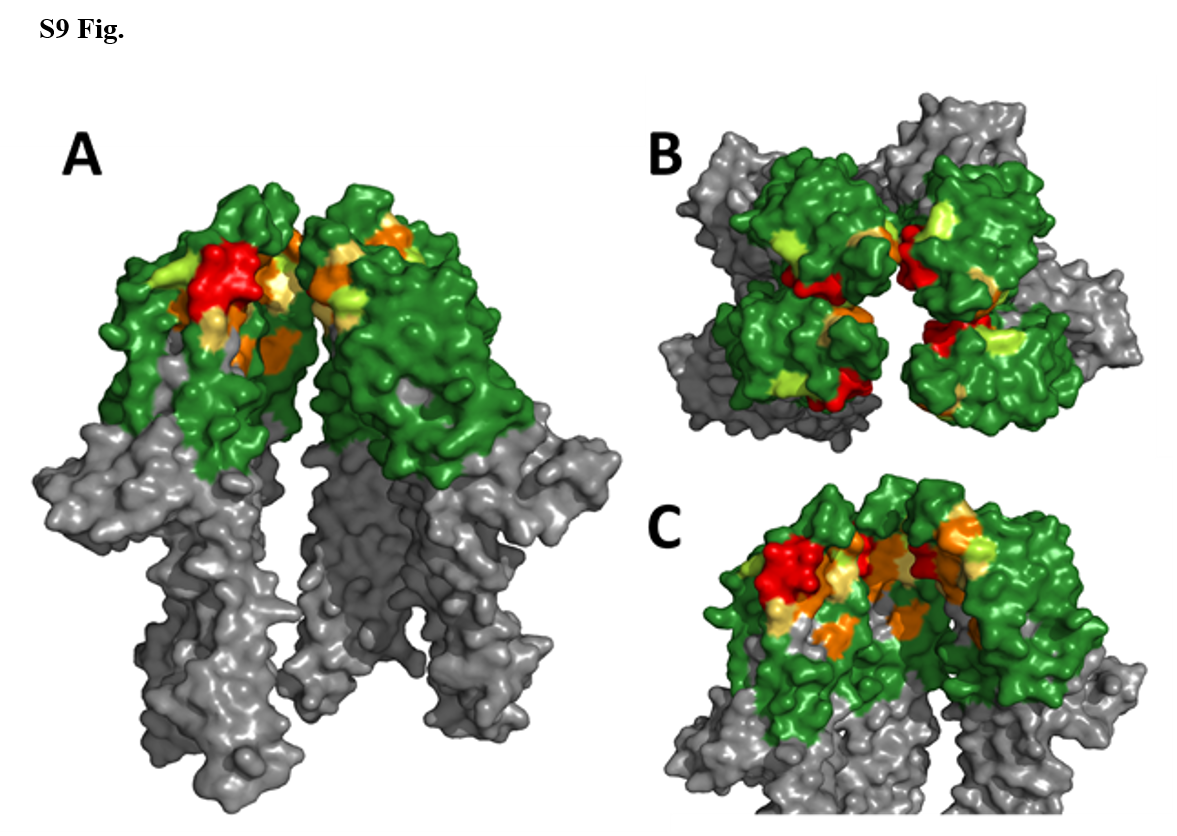

Supplement: S9 Fig — Representation of the epitope of mAb IpaD-318 on the proposed model (PDB #4d3e) for the tip complex of Blocker and co-workers. In this model, the fifth member of the pentamer is supposed to be IpaB (not represented). (A) Side view. (B) Upper View. (C) Side view focused on the epitope lacking the 4th subunit. (TIF) [file pntd.0009231.s009.tif]
