# Supplementary material for: An antibody targeting type III secretion system induces broad protection against Salmonella and Shigella infections
Source: PLoS Negl Trop Dis. 2021 Mar 12;15(3):e0009231. doi: 10.1371/journal.pntd.0009231 (PMC7990167; doi:10.1371/journal.pntd.0009231)
Supplement: S1 Text — Table A. Numbers and isotypes of IpaD/SipD monoclonal antibodies produced against IpaD protein. The isotypes were identified by a sandwich ELISA test using antibodies specific to each isotype. Although recognizing both proteins and as they were obtained from mice immunized with IpaD, they were named “IpaD”. Table B. In vivo selection of anti-IpaD/SipD monoclonal antibodies with neutralizing activity against intranasal S. flexneri 2a or intragastric S. Typhimurium challenge. Mice (N = 3) were injected twice with 500 μg of each mAb (24 hours before the challenge and 4 days later) by the intraperitoneal route and were challenged with 100 LD50 of S. Typhimurium by the intragastric route. Survival was monitored for 30 days. (DOCX) [file pntd.0009231.s010.docx]

**S1 text**

**Table A. Numbers and isotypes of IpaD/SipD monoclonal antibodies produced against IpaD protein.**


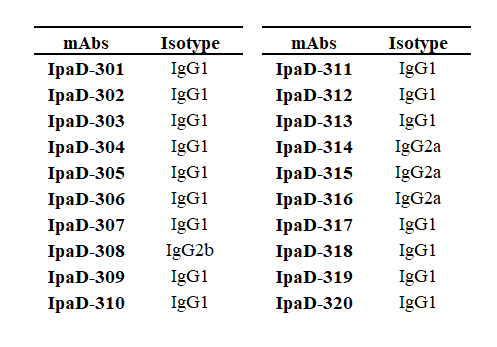


The isotypes were identified by a sandwich ELISA test using antibodies specific to each isotype. Although recognizing both proteins and as they were obtained from mice immunized with IpaD, they were named “IpaD”.

**Table B. *In vivo* selection of anti-IpaD/SipD monoclonal antibodies with neutralizing activity against intranasal *S. flexneri 2a* or intragastric *S.* Typhimurium challenge.**

| **mAb** | **Survival** | **mAb** | **Survival** | **mAb** | **Survival** | **mAb** | **Survival** |
| --- | --- | --- | --- | --- | --- | --- | --- |
| **IpaD-301** | 1/3 | **IpaD-306** | 2/3 | **IpaD-311** | 1/3 | **IpaD-316** | 1/3 |
| **IpaD-302** | 1/3 | **IpaD-307** | 1/3 | **IpaD-312** | 0/3 | **IpaD-317** | 3/3 |
| **IpaD-303** | 1/3 | **IpaD-308** | 1/3 | **IpaD-313** | 0/3 | **IpaD-318** | 2/3 |
| **IpaD-304** | 0/3 | **IpaD-309** | 0/3 | **IpaD-314** | 0/3 | **IpaD-319** | 0/3 |
| **IpaD-305** | 0/3 | **IpaD-310** | 0/3 | **IpaD-315** | 1/3 | **IpaD-320** | 0/3 |

Mice (N = 3) were injected twice with 500 μg of each mAb (24 hours before the challenge and 4 days later) by the intraperitoneal route and were challenged with 100 LD50 of *S.* Typhimurium by the intragastric route. Survival was monitored for 30 days.
